# In Vitro Antioxidant and Pancreatic Anticancer Activity of Novel 5-Fluorouracil-Coumarin Conjugates

**DOI:** 10.3390/pharmaceutics14102152

**Published:** 2022-10-10

**Authors:** Sonia López, Ignacio Gracia, Rodrigo Plaza-Pedroche, Juan Francisco Rodríguez, José Manuel Pérez-Ortiz, Julián Rodríguez-López, María Jesús Ramos

**Affiliations:** 1 Departamento de Ingeniería Química, Instituto de Tecnología Química y Medioambiental (ITQUIMA), Universidad de Castilla-La Mancha, Avda. Camilo José Cela 1A, 13071 Ciudad Real, Spain; 2 Área de Química Orgánica, Facultad de Ciencias y Tecnologías Químicas, Universidad de Castilla-La Mancha, Avda. Camilo José Cela 10, 13071 Ciudad Real, Spain; 3 Unidad de Investigación Traslacional, Hospital General Universitario de Ciudad Real, C/Obispo Rafael Torija s/n, 13005 Ciudad Real, Spain

**Keywords:** anticancer, antioxidant, click chemistry, 5-fluorouracil, coumarins, conjugates

## Abstract

Molecular hybridization consists of the combination of two or more non-identical pharmacophores in a single molecule. It has emerged as a promising strategy that allows the design of molecular frameworks with enhanced activity and affinity compared to their parent drugs. In this work, two novel hybrids that combine the well-known anticancer chemotherapeutic agent 5-fluorouracil with antioxidant coumarin derivatives have been synthesized and characterized by means of a copper-catalyzed azide-alkyne cycloaddition (CuAAC). The conjugates showed good antioxidant properties and a high tendency to aggregate and form stable nanoparticles in aqueous media, with regular shape and uniform size. These materials have proven to be preferential cytotoxic agents in vitro against human pancreatic cancer cells PANC-1, with an activity superior to free 5-fluorouracil. These results open up the possibility of exploiting the synergistic combination between 5-fluorouracil and coumarin derivatives and warrant further investigation of these hybrids as promising pancreatic anticancer agents.

## 1. Introduction

Cancers are a major public health problem and one of the main causes of death worldwide, nowadays claiming the lives of more than 9 million people a year [1]. Almost 1 in 6 deaths in the world is due to this disease. In most developed countries, cancers are the second leading cause of death after cardiovascular diseases [2]. Moreover, assuming the continuation of current trends, cancer cases are expected to increase as a result of the population ageing and the adoption of unhealthy lifestyles [3,4].

Numerous drugs have been developed to treat cancers, but the incomplete efficacy of treatments and the undesired side effects associated with current drugs are the main challenges for therapy. Today, it is well recognized that the combination of drugs with different mechanisms of action on more than a single target is usually more effective due to an enhanced synergistic, additive, and potentiating activity [5,6]. Consequently, combinational drug treatment has been adopted as a standard practice in the clinic. Nevertheless, the traditional cocktails often suffer from different pharmacokinetics for each individual drug, leading to an uncoordinated biodistribution that limits the improvement in therapeutic efficacy. Thus, new strategies are emerging for the precise and controlled delivery of multiple therapeutic agents in order to obtain successful clinical outcomes [7,8]. Molecular hybridization is a rational design strategy that has been used over the years to potentially achieve the benefits of drug combination. This molecular technique can covalently combine multiple pharmacophores into a single molecule. It has proven to be an effective tool to increase the pharmacological activity and efficacy of the bioactive constituents of the hybrid molecule and to potentially overcome the resistance to powerful antitumor agents, with reduced toxicity to other tissues [9,10,11].

In the last years, click chemistry has been established as one of the most valuable methods for linking functional moieties. In particular, copper(I)-catalyzed azide-alkyne cycloaddition (CuAAC) has been one of the most prolific and successful click reactions in different areas of life and material sciences such as drug discovery, bioconjugation applications, and surface and polymer chemistry [12,13,14]. CuAAC is a type of Huisgen 1,3-dipolar cycloaddition based on the formation of 1,4-disubstituted 1,2,3-triazoles between a terminal alkyne and an aliphatic azide in the presence of copper [15,16]. The formed 1,2,3-triazole ring is a favorable linker that displays a marked stability to metabolic degradation and oxidative/reductive conditions and actively participates in binding to biomolecular targets, significantly contributing to biological activity [12]. It is an excellent mimetic of amide bonds [17,18], although due to its high chemical stability, it is still unknown how triazoles are metabolized and more research is needed to determine their biological fate [19]. Thus, this heterocycle is a very well-recognized pharmacophore [19,20] that has been widely exploited in drug development [21]. 1,2,3-Triazole derivatives have numerous therapeutically important activities such as anticancer, antifungal, antibacterial, antitubercular, and antiviral activities [22,23,24,25].

In this work, a molecular hybridization approach is adopted by combining 5-fluorouracil (**5-FU**) with coumarin scaffolds. **5-FU** is a rationally designed analogue of the naturally occurring pyrimidine uracil that has been part of the therapeutic armamentarium for the treatment of a number of common malignancies, including colon, breast, and skin cancers [26,27]. Unfortunately, **5-FU** shows major drawbacks such as a short half-life, erratic bioavailability, and various undesired side effects [28]. Throughout its long history, different strategies have been developed to improve the clinical efficiency of **5-FU** such as the synthesis of new derivatives, combination or conjugation with other types of drugs, and entrapment or binding to polymers [29,30,31]. On the other hand, coumarin is a privileged scaffold in medicinal chemistry due to its vast pharmacological attributes [32]. Coumarin is a natural hydrophobic drug well-known for its antioxidant capacity. The main function of antioxidants is to pair off the odd electron present in free radicals via single electron or hydrogen atom transfer. Free radicals have a wide range of effects on biological systems and play an important role in the pathogenesis of certain diseases [33]. Several coumarin derivates also have antibacterial, antifungal, anticoagulant, anti-HIV, anti-inflammatory, antihypertensive, and anticancer properties [34,35]. In addition, coumarins are not only effective towards cancer but also capable of reducing the side effects induced by radiotherapy [36,37]. These anticancer benefits allow the use of coumarin in the development of promising hybrid molecules.

**Scheme 1:**
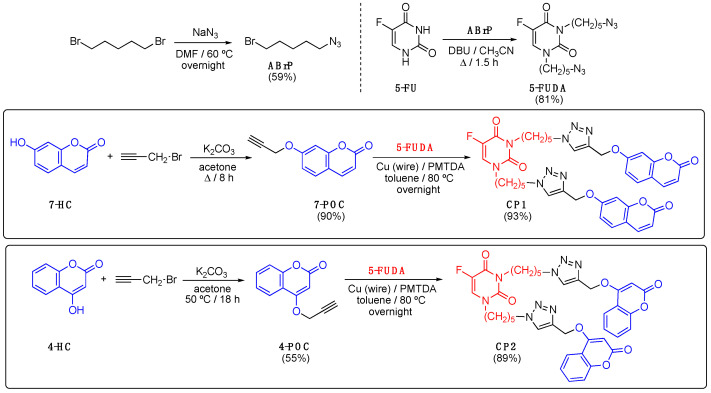
Synthesis of compounds.

The drugs were linked together via a CuAAC click reaction as shown in Scheme 1, in order to combine and synergize their aforementioned attributes. Once the synthesis was verified using a variety of analytical techniques, the antioxidant capacity of the clicked conjugates was evaluated using a DPPH (1,1-diphenyl-2-picrylhydrazyl) assay, which showed promising results. We also prepared stable nanoparticles (NPs) in aqueous media, whose shape and size were studied via high-resolution scanning electron microscopy (HR-SEM), dynamic light scattering (DLS), and zeta potential. Finally, the potential use of these materials as cytotoxic agents against human pancreatic cancer cell line PANC-1 is discussed. Similar uracil-coumarin hybrids have been previously reported as antibreast cancer and antibacterial agents, which reveals the great potential of these structures [38].

## 2. Materials and Methods

### 2.1. Materials

1,5-Dibromopentane, 5-fluorouracil, 7-hydroxycoumarin, 4-hydroxycoumarin, 1,1-diphenyl-2-picrylhydrazyl (DPPH), and pyrene were obtained from commercial sources and were used without further purification. The copper wire, 0.25 mm of diameter, was purchased from Sigma-Aldrich. 1,3-Bis(5-azidopentyl)-5-fluorouracil (**5-FUDA**) was prepared as described elsewhere by us [39]. In air- and moisture-sensitive reactions, all glassware was flame-dried and cooled under argon. All other chemicals used for this study were provided by domestic suppliers and were analytical grade unless otherwise stated.

### 2.2. Methods

NMR spectra were acquired at room temperature on a Bruker Avance Neo 500 spectrometer. The chemical shifts (δ) are reported in ppm and are referenced internally to the solvent signals of CDCl_3_ (^1^H, δ = 7.27 ppm; ^13^C, δ = 77.0 ppm) or externally to CFCl_3_ (^19^F, 0.0 ppm). The coupling constants *J* are given in Hz. In the ^1^H NMR spectra, the following abbreviations are used to describe the peak patterns: s (singlet), d (doublet), t (triplet), m (multiplet). In the ^13^C NMR spectra, the nature of the carbons (C, CH, CH_2_, or CH_3_) was determined by performing APT or DEPT experiments. The IR spectra were recorded with a Jasco FT/IR-4700 spectrophotometer equipped with an attenuated total reflectance (ATR) accessory. MALDI-TOF mass spectra were recorded on a Bruker Autoflex II spectrometer in positive detection mode, using dithranol as matrix. Melting points (°C) were measured on a Büchi M-565 apparatus and are uncorrected. Elemental analyses were performed in a Thermo Scientific Flash Smart elemental analyzer (Thermo Scientific, Waltham, MA, USA).

### 2.3. Synthesis

#### 2.3.1. 1-Azido-5-bromopentane (**ABrP**)

1-Azido-5-bromopentane was prepared according to a literature procedure [40,41]. NaN_3_ (286 mg, 4.39 mmol) was added in small portions over 2 h to a solution of 1,5-dibromopentane (1.2 mL, 8.8 mmol) in DMF (30 mL) and stirred overnight at 60 °C. After cooling, water was added and the mixture extracted with EtAcO (×3). The combined organic extracts were washed with brine, dried (MgSO_4_), filtered, and the solvent evaporated under reduced pressure. The crude product was purified via column chromatography (silica gel, hexanes to hexanes/EtAcO, 9:1). **ABrP** was obtained as a colorless liquid (495 mg, 59%). ^1^H NMR (CDCl_3_, 500 MHz) δ: 3.43 (t, 2H, *J* = 6.5 Hz, CH_2_Br), 3.31 (t, 2H, *J* = 6.5 Hz, CH_2_N_3_), 1.87–1.94 (m, 2H, CH_2_), 1.67–1.61 (m, 2H, CH_2_), 1.58–1.51 (m, 2H, CH_2_). ^13^C NMR (CDCl_3_, 125 MHz) δ: 51.2 (CH_2_N_3_), 33.3 (CH_2_Br), 32.2, 28.1, 25.3. The spectroscopy data matched with those reported in the literature.

#### 2.3.2. 7-Propargyloxycoumarin (**7-POC**)

**7-POC** was prepared according to a literature procedure [42,43]. To a solution of 7-hydroxycoumarin (umbelliferone) (1 g, 6.17 mmol) in acetone (40 mL), anhydrous potassium carbonate (1 g, 7.23 mmol) and propargyl bromide (0.88 g, 7.40 mmol) were added. The resulting mixture was stirred at reflux for 8 h. After cooling, the mixture was filtered and the solvent removed under reduced pressure. The crude product was purified via crystallization from methanol to give 1.11 g (90%) of the title compound. ^1^H NMR (CDCl_3_, 500 MHz) δ: 7.65 (d, 1H, *J* = 9.0 Hz, CH=), 7.41 (d, 1H, *J* = 8.5 Hz, ArH), 6.95 (d, 1H, *J* = 2.5 Hz, ArH), 6.92 (dd, 1H, *J* = 8.5 Hz, *J* = 2.5 Hz, ArH), 6.29 (d, 1H, *J* = 9.5 Hz, CH=), 4.77 (d, 2H, *J* = 2.5 Hz, OCH_2_), 2.58 (t, 1H, *J* = 2.5 Hz, CH≡). ^13^C NMR and APT (CDCl_3_, 125 MHz) δ: 161.0 (C), 160.5 (C), 155.6 (C), 143.2 (CH), 128.8 (CH), 113.6 (CH), 113.1 (C), 113.0 (CH), 102.1 (CH), 77.3 (C≡), 76.5 (CH≡), 56.2 (CH_2_). IR (ATR) ν: 3274 (C-H, terminal alkyne), 2116 (C≡C), 1670 (C=O), 1610, 1269, 1230, 1127, 1010, 825, 716, 636 cm^−1^. The spectroscopy data matched with those reported in the literature.

#### 2.3.3. Click Product **CP1**

A mixture of **5-FUDA** (35 mg, 0.1 mmol), **7-POC** (40 mg, 0.2 mmol), *N,N,N′,N′,N′′*-pentamethyldiethylenetriamine (PMDTA, 18 mg, 0.1 mmol), and copper wire (635 mg, 10 mmol) in toluene (10 mL) was heated to 80 °C and stirred overnight under argon atmosphere. Then, the copper was removed and the solvent evaporated under vacuum. The crude product was purified via column chromatography (silica gel, EtAcO to EtOAc/MeOH, 9:1) to give 70 mg (93%) of an oil that became a colorless solid after the addition of Et_2_O. The solid was filtered off and dried under high vacuum for several hours. Mp 66.7–67.5 °C. ^1^H NMR (CDCl_3_, 500 MHz) δ: 7.68 (s, 2H, 2×CH= triazole), 7.64 (d, 2H, *J* = 9.5 Hz, 2×CH=), 7.39 (d, 2H, *J* = 8.5 Hz, ArH), 7.22 (d, 1H, *J_H-F_* = 5.0 Hz, N-CH=CF), 6.95–6.90 (m, 4H, ArH), 6.26 (d, 1H, *J* = 9.5 Hz, CH=), 6.25 (d, 1H, *J* = 9.5 Hz, CH), 5.25 (s, 2H, OCH_2_), 5.25 (s, 2H, OCH_2_), 4.42–4.37 (m, 4H, 2×CH_2_), 3.94 (t, 2H, *J* = 7.5 Hz, CH_2_), 3.71 (t, 2H, *J* = 7.5 Hz, CH_2_), 2.03–1.96 (m, 4H, 2×CH_2_), 1.77–1.65 (m, 8H, 4×CH_2_), 1.41–1.33 (m, 4H, 2×CH_2_). ^13^C NMR and DEPT (CDCl_3_, 125 MHz) δ: 161.3 (C), 161.3 (C), 161.0 (C), 161.0 (C), 157.1 (d, *J_C-F_* = 24.8 Hz, CF-CO-NH), 155.7 (C), 149.7 (C), 143.3 (CH), 143.3 (CH), 143.1 (C), 142.9 (C), 140.0 (d, *J_C-F_* = 234.1 Hz, C-F), 128.9 (CH), 128.9 (CH), 126.5 (d, *J_C-F_* = 31.8 Hz, CH=), 123.0 (C), 113.5 (CH), 113.4 (CH), 113.0 (C), 112.9 (C), 112.8 (CH), 112.7 (CH), 102.1 (CH), 102.1 (CH), 62.3 (CH_2_), 62.3 (CH_2_), 50.1(CH_2_), 50.0 (CH_2_), 49.5 (CH_2_), 41.3 (CH_2_), 29.6 (CH_2_), 28.1 (CH_2_), 26.5 (CH_2_), 23.4 (CH_2_), 23.2 (CH_2_). ^19^F NMR (CDCl_3_, 470 MHz) δ: −164.4. IR (ATR) ν: 1724 (C=O), 1708 (C=O), 1681, 1650, 1608, 1275, 1228, 1120, 1002, 832, 758, 727 cm^−1^. MALDI-TOF MS (dithranol) *m/z*: 753.6 [M+H]^+^. Anal. Calcd for C_38_H_37_FN_8_O_8_: C, 60.63; H, 4.95; N, 14.89. Found: C, 61.29; H, 4.95, N, 15.39.

#### 2.3.4. 4-Propargyloxycoumarin (**4-POC**)

**4-POC** was synthesized according to a literature procedure [44,45]. To a solution of 4-hydroxycoumarin (1 g, 6.17 mmol) in acetone, anhydrous potassium carbonate (1 g, 6.17 mmol) and propargylbromide (0.88 g, 7.40 mmol) were added. The resulting mixture was stirred at 50 °C for 18 h. Then, the mixture was cooled and the solvent removed under reduced pressure. The residue was treated with 15 mL of water and extracted with EtAcO (×3). The combined organic extracts were dried (MgSO_4_), filtered, and the solvent evaporated under reduced pressure. The crude was purified via crystallization from a mixture of EtAcO/hexanes to give 0.68 mg (55%) of the title compound as a colorless solid. ^1^H NMR (CDCl_3_, 500 MHz) δ: 7.85 (dd, 1H, *J* = 8.0 Hz, *J* = 1.5 Hz, ArH), 7.59–7.55 (m, 1H, ArH), 7.34 (dd, 1H, *J* = 8.5 Hz, *J* = 1.0 Hz, ArH), 7.31–7.28 (m, 1H, ArH), 5.85 (s, 1H, CH=), 4.88 (d, 2H, *J* = 2.5 Hz, OCH_2_), 2.68 (t, 1H, *J* = 2.5 Hz, CH≡). ^13^C NMR and APT (CDCl_3_, 125 MHz) δ: 164.2 (C), 162.4 (C), 153.3 (C), 132.5 (CH), 124.0 (CH), 123.0 (CH), 116.7 (CH), 115.3 (C), 91.7 (CH), 77.8 (≡CH), 75.7 (≡C), 56.8 (CH_2_). IR (ATR) n: 3227 (C-H, terminal alkyne), 2125 (C≡C), 1670 (C=O), 1684, 1611, 1567, 1494, 1353, 1240, 1109, 933, 842, 687 cm^−1^. The spectroscopy data matched with those reported in the literature.

#### 2.3.5. Click Product **CP2**

A mixture of **5-FUDA** (49.33 mg, 0.14 mmol), **4-POC** (61.66 mg, 0.31 mmol), PMDTA (25 mg, 0.14 mmol), and copper wire (88.97 mg, 10 mmol) in toluene (10 mL) was heated to 80 °C and stirred overnight under argon atmosphere. Then, the copper was removed and the solvent evaporated under vacuum. The crude product was purified via column chromatography (silica gel, EtAcO to EtOAc/MeOH, 9:1) to give 94 mg (89%) of an orange oil that became a colorless solid after the addition of Et_2_O. The solid was filtered off and dried under high vacuum for several hours. Mp 69.3–71.8 °C. ^1^H NMR (CDCl_3_, 500 MHz) δ: 7.81 (dd, 1H, *J* = 8.0 Hz, *J* = 1.5 Hz, ArH), 7.80 (dd, 1H, *J* = 8.0 Hz, *J* = 1.5 Hz, ArH), 7.75 (s, 1H, CH= triazole), 7.74 (s, 1H, CH= triazole), 7.58–7.52 (m, 2H, ArH), 7.33–7.31 (m, 2H, ArH), 7.27–7.21 (m, 3H, ArH and N-CH=CF), 5.89 (s, 1H, CH=), 5.85 (s, 1H, CH=), 5.37 (s, 2H, OCH_2_), 5.35 (s, 2H, OCH_2_), 4.46–4.41 (m, 4H, 2×CH_2_), 3.97 (t, 2H, *J* = 7.5 Hz, CH_2_), 3.72 (t, 2H, *J* = 7.5 Hz, CH_2_), 2.06–1.98 (m, 4H, 2×CH_2_), 1.79–1.68 (m, 4H, 2×CH_2_), 1.43–1.35 (m, 4H, 2×CH_2_). ^13^C NMR and DEPT (CDCl_3_, 125 MHz) δ: 165.0 (C), 164.9 (C), 162.6 (C), 162.6 (C), 157.2 (d, *J_C-F_* = 24.8 Hz, CF-CO-NH), 153.3 (C), 149.8 (C), 141.5 (C), 141.4 (C), 140.0 (d, *J_C-F_* = 234.0 Hz, C-F), 132.6 (CH), 132.5 (CH), 126.6 (d, *J_C-F_* = 32.0 Hz, CH=), 123.9 (CH), 123.9 (CH), 123.5 (CH), 123.4 (CH), 123.2 (CH), 123.1 (CH), 116.8 (CH), 116.7 (CH), 115.5 (C), 115.4 (C), 91.2 (CH), 91.1 (CH), 62.6 (CH_2_), 50.2 (CH_2_), 50.1 (CH_2_), 49.5 (CH_2_), 41.3 (CH_2_), 29.6 (CH_2_), 29.5 (CH_2_), 28.1 (CH_2_), 26.5 (CH_2_), 23.4 (CH_2_), 23.2 (CH_2_). ^19^F NMR (CDCl_3_, 470 MHz) δ: −164.3. IR (ATR) ν: 1724 (C=O), 1708 (C=O), 1650, 1608, 1275, 1228, 1120, 1002, 832, 758, 727 cm^−1^. MALDI-TOF MS (dithranol) *m/z*: 751.5 [M-H]^+^. Anal. Calcd for C_38_H_37_FN_8_O_8_: C, 60.63; H, 4.95; N, 14.89. Found: C, 60.65; H, 5.68, N, 13.58.

### 2.4. In Vitro Antioxidant Activity

The antioxidant potential of the newly synthesized compounds was evaluated in vitro via their scavenging effect on DPPH radicals [46]. The hydrogen atom or electron donating ability of the compounds was measured from the bleaching of the purple-colored methanol solution of DPPH. Thus, a methanolic solution of each compound (2 mL) at five different concentrations (10, 20, 30, 40, and 50 µM) was added to a methanolic solution of DPPH (2 mL, 100 µM). The mixtures were incubated at 37 °C (approximately the corporal temperature) for 30 min in the dark. The reduction of DPPH radicals was then determined by measuring the absorbance of the resulting solutions at λ = 516 nm. The percentage of free radical scavenging activity was calculated according to Equation (1).

(1) $$\%\;\mathrm{Scavenging}=\frac{\mathrm{control}\;\mathrm{absorbance}-\mathrm{sample}\;\mathrm{absorbance}}{\mathrm{control}\;\mathrm{absorbance}}\times 100$$

The IC_50_ values (effective concentration at which radicals were scavenged by 50%) were calculated by interpolation from the linear regression analysis. Experiments at different concentrations were carried out in triplicate.

### 2.5. Preparation of **CP1** and **CP2** Nanoparticles

The solvent evaporation technique was used for the preparation of nanoparticles. Briefly, a solution of click product (5 mg) in acetone (10 mL) was stirred for 4 h at room temperature in a round-bottom flask. Then, 10 mL of Milli-Q water was added dropwise at a speed of 2 mL h^−1^ under continuous stirring. After the formation of stable emulsion, the acetone was removed under vacuum. Finally, the NPs were diluted for further experiments with Milli-Q water to a precise concentration (10^−6^–10^−1^ mg mL^−1^).

### 2.6. Determination of the Critical Aggregation Concentration (CAC)

A stock solution of pyrene was prepared at a concentration of 1 mg mL^−1^ (4.9 × 10^−3^ M) in acetone, which was further diluted to a 4.9 × 10^−4^ M solution. The pyrene solution (10 µL) was transferred to empty vials and the acetone was allowed to evaporate completely before adding 2 mL of nanoparticles solution in Milli-Q water at different concentrations. The final concentration of pyrene in each vial was maintained at 2.45 × 10^−6^ M. The samples were vigorously stirred overnight and then filtered with a 0.45 µm polytetrafluoroethylene (PTFE) filter to remove the unencapsulated dye. The emission spectra were recorded at room temperature from 350 to 500 nm at the excitation wavelength of 335 nm, using a 1 cm quartz cell. Both slits of excitation and emission were set at 2.5 nm throughout all experiments. Plotting the ratio of the fluorescence intensities at 385 nm (I_3_) and 374 nm (I_1_) against the logarithm of the concentration of each nanoparticles sample (I_385_/I_374_ vs. log C) gave a curve where the point of inflection was equal to the CAC.

### 2.7. Dynamic Light Scattering (DLS)

The apparent hydrodynamic size of the nanoparticles was obtained via the direct solution method and determined using a DLS instrument with a Zetasizer Nano ZSP of Malvern. The light source was a 10 mW He-Ne laser operating at a fixed wavelength of 633 nm, a scattering angle of 90°, and a temperature of 36 °C (the corporal temperature, approximately). The hydrodynamic diameter, D_h_, which can be defined as the diameter of a hypothetical sphere that diffuses at the same rate as the particle under investigation, was calculated using the Stokes-Einstein equation, Equation (2) [47]:

(2) $$D_{h}=\frac{k_{B}\times T}{{3\pi \times \eta \times D}_{t,\mathrm{avg}}}$$

where k_B_ is the Boltzmann coefficient (1.380 × 10^−23^ J K^−1^), T is the absolute temperature, η is the medium viscosity, and D_t,avg_ is the translational diffusion coefficient. In our case, the viscosity of the water was considered (0.001 kg m^−1^ s^−1^) due to the high dilution of the samples. The measurements were carried out at different concentrations in triplicate, and the software calculated the polydispersity index.

The zeta potential (ζ), also termed the electrokinetic potential, is the potential at the slipping/shear plane of a colloid particle moving under an electric field. The zeta potential is the key parameter that controls electrostatic interactions in particle dispersions, and as such, it is important in understanding the stability of colloidal dispersions and is very well-known to be an important indicator of the stability of nanoparticles [48]. The stability of **CP1** and **CP2** nanoparticles was analyzed via ζ measurements, using the laser Doppler microelectrophoresis technique with fitting to the Smoluchowski equation employing a Z-Sizer Nano ZS (from Malvern Instruments). This procedure allows ζ of particle suspensions to be determined with a diameter comprised within 3.8 nm to 100 μm. All the measurements were carried out in triplicate.

### 2.8. High Resolution Scanning Electron Microscopy (HR-SEM)

The morphology of the nanoparticles was determined via HR-SEM at room temperature using a ZEISS GeminiSEM 500 FE-SEM with a PIN-diode BSE detector. The average size was determined using ImageJ software. Samples were prepared via dropwise addition of the solution onto a glass plate followed by solvent evaporation in air.

### 2.9. Viability Assays: Cell Culture

The human pancreatic cancer cell lines PANC-1 and BxPC3 and the normal human control skin fibroblasts CRL-2072 were obtained from the American Type Culture Collection ((PANC-1) ATCC^®^ CRL-1469™; (BxPC3) ATCC^®^ CRL-1687™; (CCD-1059Sk) ATCC^®^ CRL-2072™). PANC-1 cells were maintained in RPMI-1640 medium (Sigma Aldrich, Darmstadt, Germany). BxPC3 and CRL-2072 were maintained in DMEM medium (Dulbecco’s modified Eagle’s medium; Sigma Aldrich, Darmstadt, Germany). All cell cultures were supplemented with 10% fetal bovine serum and antibiotics (penicillin 100 IU mL^−1^ and streptomycin 100 g mL^−1^, Sigma Aldrich, Darmstadt, Germany) and kept at 37 °C in an atmosphere of 5% CO_2_.

### 2.10. Viability Assays: MTT Assay

In vitro MTT (3-(4,5-dimethylthiazol-2-yl)-2,5-diphenyltetrazolium bromide) assays were used to assess the cytotoxic activity of **5-FU**, **5-FUDA**, **CP1** and **CP2** nanoparticles, **4-HC**, **7-HC**, **4-POC**, and **7-POC** against PANC-1, BxPC3, and CRL-2072 cells. The MTT assay is based on the conversion of MTT into formazan crystals by living cells, which determines mitochondrial activity. Thus, 10,000 cells per well were seeded in 96-well plates in DMEM (100 µL), and after allowing the cells to adhere to the bottom of the well, different concentrations of **5-FU**, **5-FUDA**, **CP1** and **CP2** nanoparticles, **4-HC**, **7-HC**, **4-POC**, and **7-POC** in DMEM (100 µL) were added. The cells were incubated at 37 °C for 24 h or 48 h and 5% CO_2_. Then, the culture medium was replaced by 100 μL of fresh DMEM containing 0.5 mg mL^−1^ of MTT and incubated for another 2.5 h. Finally, the MTT-containing medium was removed, and the purple formazan crystals were dissolved by adding 100 μL of dimethyl sulfoxide to each well. The absorbance of MTT was measured in a Dynex Spectra MR (Chantilly, VA, USA) at 570 nm with background subtracted at 650 nm. All values were normalized with respect to control wells as indicated in Equation (3).

(3) $${\mathrm{Cell}\;\mathrm{Viability}\;(\%)=(\mathrm{Absorbance}}_{\mathrm{treated}}{/\mathrm{Absorbance}}_{\mathrm{control}})\times 100$$

Each experiment was conducted in triplicate or sextuplicate, and the average data are presented.

The MTT assay is suitable for the measurement of drug sensitivity in established cell lines as well as primary cells. For dividing cells (usually cell lines), the decrease in cell number reflects cell growth inhibition and the drug sensitivity is then usually specified as the concentration of the drug that is required to achieve 50% growth inhibition as compared to the growth of the untreated control (50% inhibitory concentration, IC_50_) [49].

### 2.11. Viability Assays: Morphology Analysis—Optical Microscopy Images

The effect of the different drugs on cell morphology after 24 h incubation was studied using optical microscopy. PANC-1 morphology was imaged via optical microscopy using a Nikon ECLIPSE Ti-U microscope (Tokyo, Japan) at 10× and 20×, before the corresponding cell proliferation assay.

### 2.12. Viability Assays: Statistical Analysis

The GraphPad PRISM software 6.01 was used for the statistical analysis. Evaluations between experimental groups were performed with one-way ANOVA with multiple comparisons followed by Dunnett’s post hoc test. Statistical significance was considered at the 0.05 level.

## 3. Results and Discussion

### 3.1. Synthesis and Characterization of **CP1** and **CP2**

The synthetic routes to click products **CP1** and **CP2** are given in Scheme 1. Firstly, 1-azido-5-bromopentane (**ABrP**) was obtained from 1,5-dibromopentane and sodium azide. Subsequent reaction with 5-fluorouracil (**5-FU**) under basic conditions (DBU) yielded 1,3-bis(5-azidopentyl)-5-fluorouracil (**5-FUDA**). In parallel, coumarin derivatives **7-POC** and **4-POC** were prepared from the corresponding hydroxycoumarins (**7-HC** and **4-HC**, respectively) via treatment with propargyl bromide in presence of potassium carbonate. Finally, conjugates **CP1** and **CP2** were accessed via CuAAC using Cu wire as copper source and PMDTA as ligand. The chemical structures of **CP1** and **CP2** were fully characterized via NMR, FT-IR, MALDI-TOF mass spectrometry (Figures S1–S28 in the Supplementary Materials), and elemental analysis. The characteristic triazole protons appeared as singlets at 7.75–7.68 ppm in the ^1^H NMR spectra of **CP1** and **CP2** (Figures S13 and S23, respectively). Regarding the IR spectra, the typical azide band of **5-FUDA** (2089 cm^−1^, Figure S7) and the terminal acetylene bands of **7-POC** (3274 and 2116 cm^−1^, Figure S12) and **4-POC** (3227 and 2125 cm^−1^, Figure S22) are not observed in the spectra of **CP1** and **CP2** (Figures S17 and S27, respectively), confirming the successful synthesis of the click conjugates.

### 3.2. In Vitro Antioxidant Evaluation

The antioxidant activities of the **CP1** and **CP2** conjugates, as well as the precursor coumarin derivatives, have been assessed in vitro by means of the DPPH radical scavenging assay. Ascorbic acid was employed as a reference standard. The results expressed as mean ± SD (standard deviation), along with IC_50_ values obtained via regression analysis, are summarized in Table 1. All compounds were tested at five different concentrations of 10, 20, 30, 40, and 50 μM, exhibiting significant dose-dependent scavenging activities that ranged from 48.37 to 90.95% compared to the standard ascorbic acid (45.78–95.42%). It is worth noting that both conjugates exhibited a higher antioxidant activity (IC_50_ 2.82 and 5.38 μM, respectively) than the standard (IC_50_ = 11.42 μM). **CP1** showed a lower IC_50_ than **CP2** even though the IC_50_ for precursor **7-HC** was higher than that of **4-HC** (13.82 vs. 1.55 μM).

### 3.3. Preparation and Characterization of CP1 and CP2 Nanoparticles

**Figure 1:**
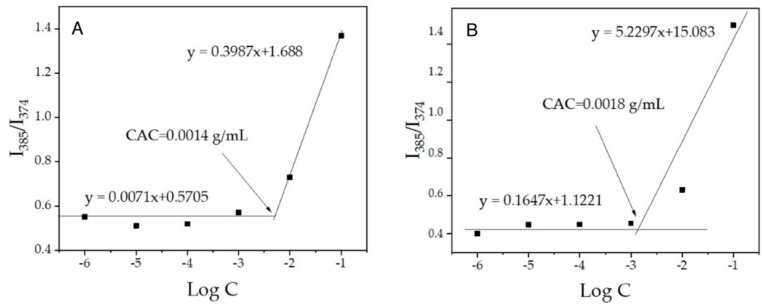
Measurement of the CAC via the pyrene fluorescence probe method; (**A**) **CP1** and (**B**) **CP2**.

The amphiphilic hybrids **CP1** and **CP2** could self-assemble and form nanoparticles in aqueous solution, presumably with the hydrophobic coumarin moieties located in the inner part of the particles and the hydrophilic **5-FU** and 1,2,3-triazole groups in the outer part. The NPs were prepared using the solvent evaporation technique, and the critical aggregation concentration (CAC) was determined using the pyrene fluorescence probe method [50]. The data obtained confirmed that both click products exhibited a high tendency to aggregation, even at relatively low concentrations with a CAC of 0.0014 mg·mL^−1^ (1.8 mM) for **CP1** and 0.0018 mg mL^−1^ (2.3 mM) for **CP2** in Milli-Q water (Figure 1).

**Figure 2:**
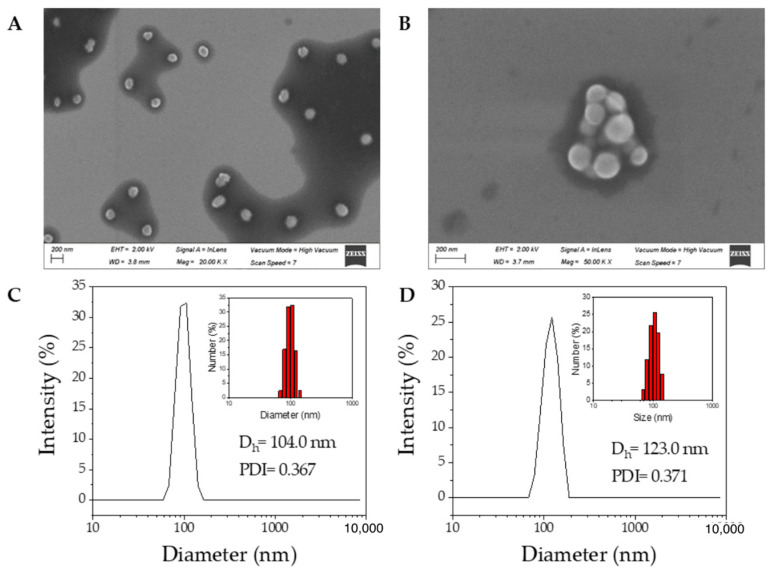
(**A**,**B**) HR-SEM images of **CP1** and **CP2** NPs, respectively. (**C**,**D**): DLS analysis of **CP1** and **CP2** NPs, respectively (c = 0.1 mg mL^−1^). The corresponding histograms are shown in the insets.

The average particle size and size distribution are also important parameters for drug self-delivery systems. The hydrodynamic size and size distribution of the **CP1** and **CP2** NPs were evaluated via DLS at a concentration higher than the CAC, i.e., 0.1 mg mL^−1^, and the morphology was determined via HR-SEM, as shown in Figure 2. The HR-SEM showed a spherical morphology for the NPs with average sizes of 114.34 ± 34.62 nm for **CP1** and 93.19 ± 28.12 nm for **CP2** (Figure 2A,B). On the other hand, the DLS analysis revealed a monomodal size distribution peak with a *z*-averaged diameter of 104.0 nm as well as a calculated polydispersity index (PDI) of 0.367 for **CP1** and 123.0 nm with PDI 0.371 for **CP2** (Figure 2C,D). As expected, the data from HR-SEM images do not exactly match with the information received from DLS, although both results are consistent. DLS is an intensity-based technique while HR-SEM is a number-based technique, which makes them fundamentally different [51].

Additionally, DLS and zeta potential measurements were carried out at a range of time intervals to evaluate the stability of the nanoparticles (1–18 days). The results are summarized in Table 2, and they reveal that the **CP1** and **CP2** NPs remained stable for at least one week. The colloids lost stability and agglomerated/flocculated from day 9 for **CP1** and day 7 for **CP2** since they were closer to the isoelectric point, i.e., the point where the zeta potential is equal to zero.

### 3.4. Preliminary Efficacy Assessment in Cell Culture: Cell Viability

**Figure 3:**
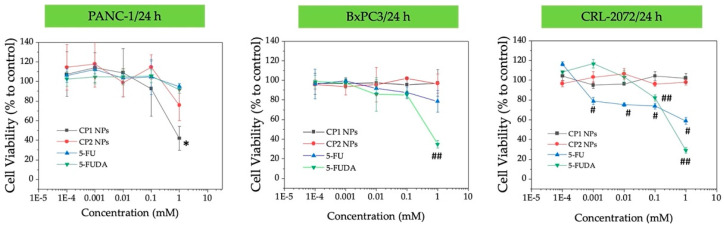
Cell viability of PANC-1, BxPC3, and CRL-2072 cells treated with **CP1** and **CP2** NPs, **5-FU**, and **5-FUDA** at different doses (from 0.0001 mM to 1 mM) after 24 h of incubation time. Values represent means of three to six replicates ± SD of one representative experiment. * *p* < 0.05, **CP1** NPs group. # *p* < 0.05, **5-FU** group. ## *p* < 0.05, **5-FUDA** group.

The cell viability of pancreatic cancer PANC-1 and BxPC3 cells, compared to that of normal human control fibroblasts CRL-2072, was evaluated via treatment with different doses of **5-FU, 5-FUDA**, and **CP1** and **CP2** NPs for 24 h through an in vitro MTT assay. Figure 3 shows that all compounds promoted cell proliferation (hormetic effect) at concentrations lower than 0.01 mM in PANC-1 and CRL-2072. Hormesis is widely found in a great variety of chemotherapeutic agents (included **5-FU**) and natural compounds isolated from plants [52,53,54]. Nevertheless, at concentrations higher than 0.1 mM, cells gradually decreased their viability with the increase of drug concentration, indicating a dose-dependent antiproliferative activity. **CP1** and **CP2** NPs were more cytotoxic than **5-FU** over the dose range 0.0001–1 mM on PANC-1 but did not increase cell demise on BxPC3 and CRL-2072. In this latter cell line, only **5-FU** and **5-FUDA** were able to significantly decrease cell viability. In order to characterize the efficacy of the drugs, the maximum inhibitory concentration (IC_50_) for PANC-1 was also determined. The IC_50_ ± SD values of **5-FUDA**, **CP1**, and **CP2** fitted to an exponential equation were calculated using Excel software (Microsoft). The mean IC_50_ ± SD values were 5.5061 ± 0.003 mM, 0.7699 ± 0.0021 mM, and 2.2841 ± 0.0085 mM, respectively. Nevertheless, the IC_50_ for **5-FU** could not be determined because the cell viability was greater than 50%, which is in line with previous work [55,56]. According to the literature, the IC_50_ value for **5-FU** after incubation for 48 h with PANC-1 cells (37 °C, 5% CO_2_) was 12.66 μM [57].

**Figure 4:**
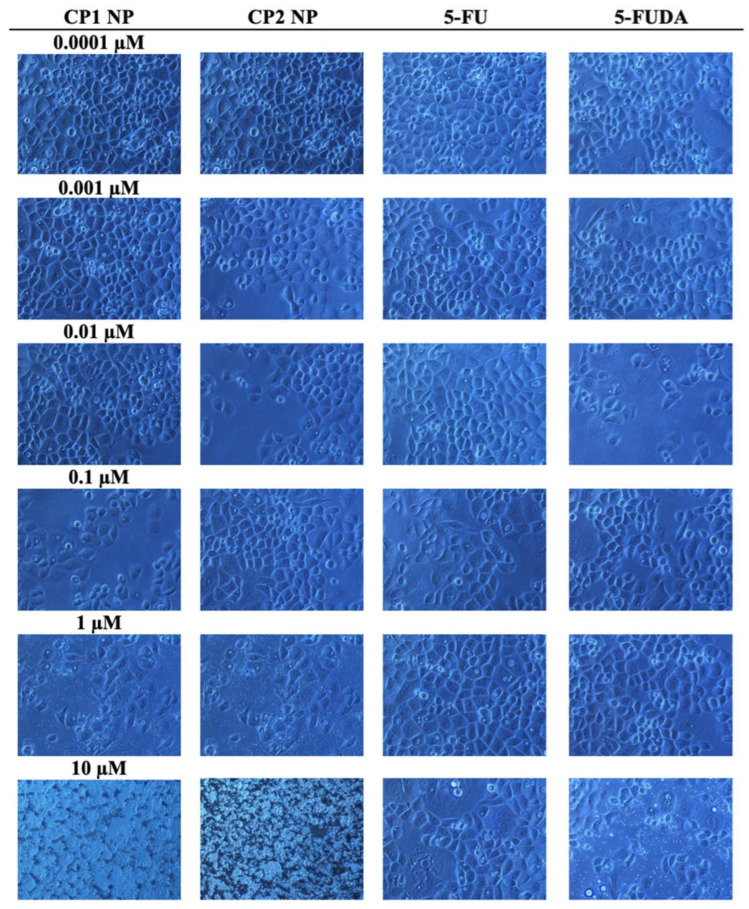
Microscopic images (magnification 20×) of PANC-1 cells after 24 h treatment with **5-FU**, **5-FUDA**, and **CP1** and **CP2** NPs in MTT assays at different concentrations.

**5-FU** and **5-FUDA** only induced a 5–8% decrease in PANC-1 cell survival at the highest dose tested (1 mM). In contrast, **5-FUDA** significantly affected the survival of BxPC3 at 1 mM, and **5-FU** and **5-FUDA** elicited a remarkable decrease in CRL-2072 cell viability, enhancing the cytotoxic effect. On the other hand, **CP1** and **CP2** NPs were more cytotoxic than **5-FU** and **5-FUDA** in PANC-1. Moreover, **CP1** NPs exhibited a lower IC_50_ value than **CP2** NPs, which indicates a better antiproliferative effect. Figure 4 and  Figure S29 (Supplementary Materials) show the observed morphological changes and cell demise, which are consistent with the dose-dependent outcome of the MTT assay.

**Figure 5:**
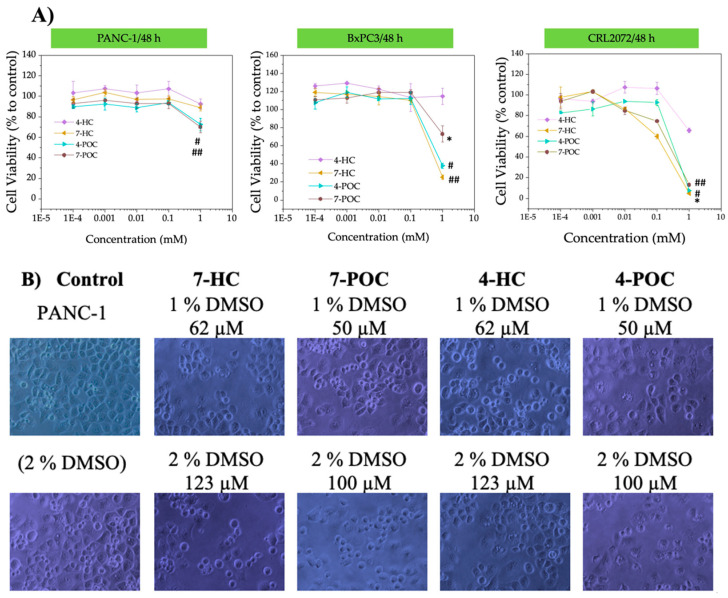
(**A**) Cell viability of PANC-1, BxPC3, and CRL-2072 cells treated with **4-HC**, **7-HC**, **4-POC**, and **7-POC** at different doses (from 0.0001 µM to 1 µM, in a solution of water with 1–2% DMSO) after 48 h of incubation (MTT assay). Values represent means of three replicates ± SD of one representative experiment. * *p* < 0.05, **7-HC** group. # *p* < 0.05, **4-POC** group. ## *p* < 0.05, **7-POC** group. (**B**) Microscopic images (magnification 20×) of the PANC-1 cells after treatment.

An additional MTT assay study was also conducted for the coumarins derivatives as they are natural compounds with pharmacological properties, including known chemotherapeutic activity. Due to their poor solubility in water, the MTT tests had to be carried out with 1% or 2% DMSO as cosolvent (Figure 5). The effect of a series of hydroxycoumarin derivatives against the human pancreatic cancer cell line PANC-1 had been assessed in previous works. Specifically, **7-HC** was tested in vitro for its preferential cytotoxicity on PANC-1 cells and no appreciable cytotoxicity was detected, even at 200 µM [58]. Nevertheless, various 3-substituted 4-anilino-coumarins have shown promising results as antiproliferative drugs on MCF-7, HepG2, HCT116, and PANC-1 cancer cell lines [59]. Some 4-(1,2,3-triazol-1-yl)coumarin conjugates have also exhibited antiproliferative activities in vitro against human breast carcinoma MCF-7 cells, colon carcinoma SW480 cells, and lung carcinoma A549 cells [60].

In this work, significant toxicity was detected for the maximum tested concentration dose (1 mM). Thus, **4-POC** and **7-POC** showed a higher percentage of cytotoxicity than **4-HC** and **7-HC** in all cell lines (Figure 5), which helps us to understand why **CP1** NPs have a greater effect against PANC-1 cells than **CP2** NPs. Moreover, **7-HC** was also able to induce a higher loss of cell viability in BxPC3 and CRL-2072 at 1 mM.

## 4. Conclusions

The synthesis of two novel triazole-linked 5-fluorouracil-coumarin conjugates, **CP1** and **CP2**, were developed via a CuAAC reaction. DPPH radical scavenging assays showed better antioxidant activity for both conjugates than that of the reference standard ascorbic acid. Due to their amphiphilic features, they can self-assemble into nanoparticles with regular shape, uniform size, and good stability in aqueous media. The pyrene fluorescence probe method, HR-SEM, and DLS measurements allowed the CAC and the size of the aggregates to be determined. The potential of these materials as cytotoxic agents was tested against human pancreatic cancer cell lines PANC-1 and BxPC3 compared to normal human control fibroblasts CRL-2072. The results of the MTT assay demonstrated that **CP1** and **CP2** nanoparticles exhibit higher cytotoxicity in PANC-1 (IC_50_ = 0.77 mM and 2.28 mM, respectively) than **5-FU** (IC_50_ = 5.51 mM) after 24 h of incubation. The presence of a 7-propargyloxycoumarin residue in the hybrid structure appears to lead to a greater effect than the presence of a 4-propargyloxycoumarin moiety. These results should enable the development of new and powerful pancreatic anticancer agents based on 5-fluorouracil and coumarin derivatives after suitable design of the molecules.

## Figures and Tables

**Table 1:** Antioxidant capacity (%) of the synthesized compounds (DPPH method) ^a^.

Compound	10 µM	20 µM	30 µM	40 µM	50 µM	IC_50_ (µM)
**7-HC**	50.42 ± 0.23	51.08 ± 0.32	52.67 ± 0.08	50.32 ± 0.16	67.39 ± 0.04	13.82 ± 0.06
**7-POC**	48.37 ± 0.12	48.53 ± 0.04	49.36 ± 0.04	50.04 ± 0.42	50.33 ± 0.03	2.95 ± 0.15
**CP1**	52.28 ± 0.30	53.6. ± 0.12	55.17 ± 0.24	55.31 ± 0.75	56.80 ± 0.46	2.82 ± 0.32
**4-HC**	71.98 ± 0.01	79.98 ± 0.01	85.42 ± 0.06	88.07 ± 0.04	90.95 ± 0.01	1.55 ± 0.01
**4-POC**	59.71 ± 0.44	60.73 ± 0.12	61.82 ± 0.07	62.38 ± 0.05	63.07 ± 0.17	1.34 ± 0.25
**CP2**	59.14 ± 0.14	69.48 ± 0.67	75.38 ± 0.23	79.30 ± 0.05	83.15 ± 0.59	5.38 ± 0.38
**Ascorbic acid**	45.78 ± 0.37	67.73 ± 0.29	78.91 ± 0.98	87.45 ± 0.41	95.42 ± 0.45	11.42 ± 0.17

^a^ Results are expressed as mean values ± SD (standard deviation), *n* = 3, *p* < 0.05.

**Table 2:** Influence of storage on diameter, PDI, and zeta potential of **CP1** and **CP2** NPs with the extension of time in water ^a^.

Time (Days)	CP1 NPs			CP2 NPs		
	DLS (nm)	PDI	ζ (mV)	DLS (nm)	PDI	ζ (mV)
1	104.0 ± 15.4	0.367	−31.8 ± 3.0	123.0 ± 4.0	0.371	−30.6 ± 3.2
2	137.8 ± 9.3	0.546	−30.1 ± 3.6	129.2 ± 1.9	0.436	−30.4 ± 3.6
3	117.4 ± 82.3	0.506	−31.8 ± 3.4	110.4 ± 14.0	0.473	−30.8 ± 3.7
4	201.9 ± 46.7	0.733	−30.1 ± 3.5	131.9 ± 4.3	0.539	−30.2 ± 3.2
5	117.3 ± 17.9	0.899	−31.8 ± 4.7	124.8 ± 29.7	0.496	−30.6 ± 3.0
6	210.5 ± 60.3	0.556	−30.1 ± 5.5	115.3 ± 24.3	0.543	−31.1 ± 2.0
7	178.3 ± 19.1	0.369	−31.8 ± 3.0	234.1 ± 20.2	1.000	−9.78 ± 3.6
8	104.6 ± 92.4	0.437	−30.1 ± 3.8	299.5 ± 25.7	1.000	−9.91 ± 3.0
9	228.0 ± 36.9	0.522	−15.0 ± 4.1	368.9 ± 70.7	0.872	−9.89 ± 3.6
10	380.4 ± 67.2	0.553	−17.2 ± 4.7	360.1 ± 104.8	0.502	−3.72 ± 3.0
11	394.9 ± 76.4	0.553	−14.5 ± 5.0	304.6 ± 38.3	0.308	−3.90 ± 3.3
12	381.0 ± 70.6	0.492	−7.04 ± 5.8	405.3 ± 58.9	0.388	−3.76 ± 3.5
13	321.6 ± 80.6	0.624	−12.1 ± 3.0	317.1 ± 59.1	0.588	−13.2 ± 3.9
14	214.9 ± 95.8	0.424	−11.3 ± 5.1	311.8 ± 186.9	0.588	−12.6 ± 4.0
15	248.4 ± 98.2	0.620	−11.7 ± 2.6	597.7 ± 234.1	0.533	−12.5 ± 3.4
16	469.2 ± 96.7	0.490	−3.69 ± 5.1	609.9 ± 215.0	0.385	−4.41 ± 2.9
17	304.6 ± 130.7	0.423	−2.92 ± 3.6	683.1 ± 321.7	0.340	−4.03 ± 3.2
18	405.3 ± 201.8	0.608	−3.69 ± 4.3	661.4 ± 178.3	0.416	−4.07 ± 2.8

^a^ Concentration = 0.1 mg mL^−1^.

## Data Availability

The data presented in this study are available within the article and Supplementary Materials.

## Supplementary Materials

The following are available online at https://www.mdpi.com/article/10.3390/pharmaceutics14102152/s1, Figure S1: ^1^H NMR of **ABrP**, Figure S2: ^13^C NMR of **ABrP**. Figure S3: ^1^H NMR of **5-FUDA**. Figure S4: ^13^C NMR of **5-FUDA**. Figure S5: DEPT of **5-FUDA**. Figure S6: ^19^F NMR of **5-FUDA**. Figure S7: FT-IR of **5-FUDA**. Figure S8: MALDI-TOF MS of **5-FUDA**. Figure S9: ^1^H NMR of **7-POC**. Figure S10: ^13^C NMR of **7-POC**. Figure S11: APT of **7-POC**. Figure S12: FT-IR of **7-POC**. Figure S13: ^1^H NMR of **CP-1**. Figure S14: ^13^C NMR of **CP-1**. Figure S15: DEPT of **CP-1**. Figure S16: ^19^F NMR of **CP-1**. Figure S17: FT-IR of **CP-1**. Figure S18: MALDI-TOF MS of **CP-1**. Figure S19: ^1^H NMR of **4-POC**. Figure S20: ^13^C NMR of **4-POC**. Figure S21: APT of **4-POC**. Figure S22: FT-IR of **4-POC**. Figure S23: ^1^H NMR of **CP-2**. Figure S24: ^13^C NMR of **CP-2**. Figure S25: DEPT of **CP-2**. Figure S26: ^19^F NMR of **CP-2**. Figure S27: FT-IR of **CP-2**. Figure S28: MALDI-TOF MS of **CP-2**. Figure S29: Microscopic images (magnification 10×) of PANC-1 cells in MTT assays.

## References

[1] World Health Organization (WHO) (2018). Latest Global Cancer Data: Cancer Burden Rises to 18.1 Million New Cases and 9.6 Million Deaths in 2018.

[2] Stewart B.W., Wild C.P. (2014). World Cancer Report 2014.

[3] Nagai H., Kim Y.H. (2017). Cancer prevention from the perspective of global cancer burden patterns. J. Thorac. Dis..

[4] Sung H., Ferlay J., Siegel R.L., Laversanne M., Soerjomataram I., Jemal A., Bray F. (2021). Global cancer statistics 2020: GLOBOCAN estimates of incidence and mortality worldwide for 36 cancers in 185 countries. CA Cancer J. Clin..

[5] Gilad Y., Gellerman G., Lonard D.M., O’Malley B.W. (2021). Drug combination in cancer treatment—From cocktails to conjugated combinations. Cancers.

[6] Hu Q., Sun W., Wang C., Gu Z. (2016). Recent advances of cocktail chemotherapy by combination drug delivery systems. Adv. Drug Deliv. Rev..

[7] Senapati S., Mahanta A.K., Kumar S., Maiti P. (2018). Controlled drug delivery vehicles for cancer treatment and their performance. Signal Transduct. Target Ther..

[8] Patra J.K., Das G., Fraceto L.F., Ramos Campos E.V., Rodríguez-Torres M.P., Acosta-Torres L.S., Diaz-Torres L.A., Grillo R., Swamy M.K., Sharma S. (2018). Nano based drug delivery systems: Recent developments and future prospects. J. Nanobiotechnol..

[9] Nepali K., Sharma S., Sharma M., Bedi P.M.S., Dhar K.L. (2014). Rational approaches, design strategies, structure activity relationship and mechanistic insights for anticancer hybrids. Eur. J. Med. Chem..

[10] Soltan O.M., Shoman M.E., Abdel-Aziz S.A., Narumi A., Konno H., Abdel-Aziz M. (2021). A five-year survey on structures of multiple targeted hybrids of protein kinase inhibitors for cancer therapy. Eur. J. Med. Chem..

[11] Dong S., He J., Sun Y., Li D., Li L., Zhang M., Ni P. (2019). Efficient click synthesis of a protonized and reduction-sensitive amphiphilic small-molecule prodrug containing camptothecin and gemcitabine for a drug self-delivery system. Mol. Pharm..

[12] Hou J., Liu X., Shen J., Zhao G., Wang P.G. (2012). The impact of click chemistry in medicinal chemistry. Expert Opin. Drug Discov..

[13] Yi G., Son J., Yoo J., Park C., Koo H. (2018). Application of click chemistry in nanoparticle modification and its targeted delivery. Biomater. Res..

[14] Mashayekh K., Shiri P. (2019). An overview of recent advances in the applications of click chemistry in the synthesis of bioconjugates with anticancer activities. ChemistrySelect.

[15] Neumann S., Biewend M., Rana S., Binder W.H. (2020). The CuAAC: Principles, homogeneous and heterogeneous catalysts, and novel developments and applications. Macromol. Rapid Commun..

[16] Nebra N., García-Álvarez J. (2020). Recent progress of Cu-catalyzed azide-alkyne cycloaddition reactions (CuAAC) in sustainable solvents: Glycerol, deep eutectic solvents, and aqueous media. Molecules.

[17] Rečnik L.-M., Kandioller W., Mindt T.L. (2020). 1,4-Disubstituted 1,2,3-triazoles as amide bond surrogates for the stabilisation of linear peptides with biological activity. Molecules.

[18] Agouram N., El Hadrami E.M., Bentama A. (2021). 1,2,3-Triazoles as biomimetics in peptide science. Molecules.

[19] Massarotti A., Aprile S., Mercalli V., Del Grosso E., Grosa G., Sorba G., Tron G.C. (2014). Are 1,4- and 1,5-disubstituted 1,2,3-triazoles good pharmacophoric groups?. ChemMedChem.

[20] Agalave S.G., Maujan S.R., Pore V.S. (2011). Click chemistry: 1,2,3-triazoles as pharmacophores. Chem. Asian J..

[21] Serafini M., Pirali T., Tron G.C. (2020). Click 1,2,3-triazoles in drug discovery and development: From the flask to the clinic?. Adv. Heterocycl. Chem..

[22] Jain A., Piplani P. (2019). Exploring the chemistry and therapeutic potential of triazoles: A comprehensive literature. Mini Rev. Med. Chem..

[23] Slanova K., Todorov L., Belskaya N.P., Palafox M.A., Kostova I.P. (2020). Developments in the application of 1,2,3-triazoles in cancer treatment. Recent Pat. Anticancer Drug Discov..

[24] Shaikh M.H., Subhedar D.D., Nawale L., Sarkar D., Khan F.A.K., Sangshetti J.N., Shingate B.B. (2015). 1,2,3-Triazole derivatives as antitubercular agents: Synthesis, biological evaluation and molecular docking study. MedChemComm.

[25] Chatterjee S., Kumar N., Sehrawat H., Yadav N., Mishra V. (2021). Click triazole as a linker for drug repurposing against SARs-CoV-2: A greener approach in race to find COVID-19 therapeutic. Curr. Res. Green Sustain. Chem..

[26] Grem J.L. (2000). 5-Fluorouracil: Forty-plus and still ticking. A review of its preclinical and clinical development. Investig. New Drugs.

[27] Pałasz A., Cież D. (2015). In search of uracil derivatives as bioactive agents. Uracils and fused uracils: Synthesis, biological activity and applications. Eur. J. Med. Chem..

[28] Diasio R.B., Harris B.E. (1989). Clinical pharmacology of 5-fluorouracil. Clin. Pharmacokinet..

[29] O’Connor O.A., Schwartz G.K. (2005). Pharmacological Modulation of Fluoropyrimidines: Building on the Lessons of the Past. Combination Cancer Therapy: Modulators and Potentiators.

[30] Almahdi E., Mohammadi-Samani S., Tayebi L., Farjadin F. (2020). Recent advances in designing 5-fluorouracil delivery systems: A stepping stone in the safe treatment of colorectal cancer. Int. J. Nanomed..

[31] Ciaffaglione V., Modica M.N., Pittalà V., Romeo G., Salerno L., Intagliata S. (2021). Mutual prodrugs of 5-fluorouracil: From a classic chemotherapeutic agent to novel potential anticancer drugs. ChemMedChem.

[32] Stefanachi A., Leonetti F., Pisani L., Catto M., Carotti A. (2018). Coumarin: A natural, privileged and versatile scaffold for bioactive compounds. Molecules.

[33] Phaniendra A., Jestadi D.B., Periyasamy L. (2015). Free Radicals: Properties, sources, targets, and their implication in various diseases. Indian J. Clin. Biochem..

[34] Venugopala K.N., Rashmi V., Odhav B. (2013). Review on natural coumarin lead compounds for their pharmacological activity. Biomed. Res. Int..

[35] Detsi A., Kontogiorgis C., Hadjipavlou-Litina D. (2017). Coumarin derivatives: An updated patent review (2015–2016). Expert Opin. Ther. Pat..

[36] Akkol E.K., Genç Y., Karpuz B., Sobarzo-Sánchez E., Capasso R. (2020). Coumarins and coumarin-related compounds in pharmacotherapy of cancer. Cancers.

[37] Yasueda A., Urushima H., Ito T. (2016). Efficacy and interaction of antioxidant supplements as adjuvant therapy in cancer treatment: A systematic review. Integr. Cancer Ther..

[38] Sanduja M., Gupta J., Singh H., Pagare P.P., Rana A. (2020). Uracil-coumarin based hybrid molecules as potent anti-cancer and anti-bacterial agents. J. Saudi Chem. Soc..

[39] López S., Rodríguez-López J., García M.T., Rodríguez J.F., Pérez-Ortiz J.M., Ramos M.J., Gracia I. (2022). Self-assembled coumarin- and 5-fluorouracil-PEG micelles as multifunctional drug delivery systems. J. Drug Deliv. Sci. Technol..

[40] Gerken P.A., Wolstenhulme J.R., Tumber A., Hatch S.B., Zhang Y., Müller S., Chandler S.A., Mair B., Li F., Nijman S.M.B. (2017). Discovery of a highly selective cell-active inhibitor of the histone lysine demethylases KDM2/7. Angew. Chem. Int. Ed..

[41] Caldarelli S.A., El Fangour S., Wein S., Tran van Ba C., Périgaud C., Pellet A., Vial H.J., Peyrottes S. (2013). New bis-thiazolium analogues as potential antimalarial agents: Design, synthesis, and biological evaluation. J. Med. Chem..

[42] Montanari S., Scalvini L., Bartolini M., Belluti F., Gobbi S., Andrisano V., Ligresti A., Di Marzo V., Rivara S., Mor M. (2016). Fatty acid amide hydrolase (FAAH), acetylcholinesterase (AChE), and butyrylcholinesterase (BuChE): Networked targets for the development of carbamates as potential anti-Alzheimer’s disease agents. J. Med. Chem..

[43] Kosiova I., Kovackova S., Kois P. (2007). Synthesis of coumarin–nucleoside conjugates via Huisgen 1,3-dipolar cycloaddition. Tetrahedron.

[44] Prasad Rao C., Srimannarayana G. (1990). Claisen rearrangement of 4-propargloxycoumarins: Formation of 2*H*,5*H*-pyrano [3,2-c][1]benzopyran-5-ones. Synth. Commun..

[45] Shaikh M.H., Subhedar D.D., Shingate B.B., Kalam Khan F.A., Sangshetti J.N., Khedkar V.M., Nawale L., Sarkar D., Navale G.R., Shinde S.S. (2016). Synthesis, biological evaluation and molecular docking of novel coumarin incorporated triazoles as antitubercular, antioxidant and antimicrobial agents. Med. Chem. Res..

[46] Wafa N., Sofiane G. (2020). In-Vitro antioxidant and anti-inflammatory activities valorisation of tannin crude extract of *Helianthemum helianthemoïdes* (Desf.) Grosser. J. Drug. Deliv. Ther..

[47] Kaszuba M., McKnight D., Connah M.T., McNeil-Watson F.K., Nobbmann U. (2008). Measuring sub nanometre sizes using dynamic light scattering. J. Nanoparticle Res..

[48] Kaszuba M., Corbett J., Watson F.M., Jones A. (2010). High-concentration zeta potential measurements using light-scattering techniques. Philos. Trans. R. Soc. A.

[49] van Meerloo J., Kaspers G.J.L., Cloos J., Cree I.A. (2011). Cell Sensitivity Assays: The MTT Assay. Cancer Cell Culture: Methods and Protocols.

[50] Aguiar J., Carpena P., Molina-Bolívar J.A., Carnero Ruiz C. (2003). On the determination of the critical micelle concentration by the pyrene 1:3 ratio method. J. Colloid Interface Sci..

[51] Bhattacharjee S. (2016). DLS and zeta potential—What they are and what they are not?. J. Control. Release.

[52] Calabrese E.J. (2005). Cancer biology and hormesis: Human tumor cell lines commonly display hormetic (biphasic) dose responses. Crit. Rev. Toxicol..

[53] Calabrese E.J. (2013). Hormetic mechanisms. Crit. Rev. Toxicol..

[54] Alley M.C., Paculacox C.M., Hursey M.L., Rubinstein L.R., Boyd M.R. (1991). Morphometric and colorimetric analyses of human tumor-cell line growth and drug sensitivity in soft agar culture. Cancer Res..

[55] Lee J.H., Lee H.-B., Jung G.O., Oh J.T., Park D.E., Chae K.M. (2013). Effect of quercetin on apoptosis of PANC-1 cells. J. Korean Surg. Soc..

[56] Wang W., Liu B., Sun S., Lan L., Chen Y., Han S., Li X., Li Z. (2020). Downregulation of miR-486-5p enhances the anti-tumor effect of 5-fluorouracil on pancreatic cancer cells. Onco. Targets Ther..

[57] El-Mahdy H.A., El-Husseiny A.A., Kandil Y.I., Gamal El-Din A.M. (2020). Diltiazem potentiates the cytotoxicity of gemcitabine and 5-fluorouracil in PANC-1 human pancreatic cancer cells through inhibition of P-glycoprotein. Life Sci..

[58] Devji T., Reddy C., Woo C., Awale S., Kadota S., Carrico-Moniz D. (2011). Pancreatic anticancer activity of a novel geranylgeranylated coumarin derivative. Bioorg. Med. Chem. Lett..

[59] Luo G., Muyaba M., Lyu W., Tang Z., Zhao R., Xu Q., You Q., Xiang H. (2017). Design, synthesis and biological evaluation of novel 3-substituted 4-anilino-coumarin derivatives as antitumor agents. Bioorg. Med. Chem. Lett..

[60] Zhang W., Li Z., Zhou M., Wu F., Hou X., Luo H., Liu H., Han X., Yan G., Ding Z. (2014). Synthesis and biological evaluation of 4-(1,2,3-triazol-1-yl)coumarin derivatives as potential antitumor agents. Bioorg. Med. Chem. Lett..

