# Supplementary material for: In Vitro Antioxidant and Pancreatic Anticancer Activity of Novel 5-Fluorouracil-Coumarin Conjugates
Source: Pharmaceutics. 2022 Oct 10;14(10):2152. doi: 10.3390/pharmaceutics14102152 (PMC9607493; doi:10.3390/pharmaceutics14102152)
Supplement: Supplementary file 1 [file pharmaceutics-14-02152-s001.zip › pharmaceutics-1968068-supplementary.pdf]

# Supplementary Materials

---

# *In vitro* antioxidant and pancreatic anticancer activity of novel 5-fluorouracil-coumarin conjugates

Sonia López,[†] Ignacio Gracia,[†] Rodrigo Plaza-Pedroche,[‡] Juan Francisco Rodríguez,[†] José Manuel Pérez-Ortiz,[§] Julián Rodríguez-López, [‡]* and María Jesús Ramos[†]*

[†] Universidad de Castilla-La Mancha, Instituto de Tecnología Química y Medioambiental (ITQUIMA), Departamento de Ingeniería Química, Avda. Camilo José Cela 1A, 13071 Ciudad Real, Spain.

[‡] Universidad de Castilla-La Mancha, Facultad de Ciencias y Tecnologías Químicas, Área de Química Orgánica, Avda. Camilo José Cela, 10, 13071 Ciudad Real, Spain.

[§] Hospital General Universitario de Ciudad Real, Unidad de Investigación Traslacional, C/ Obispo Rafael Torija s/n, 13005 Ciudad Real, Spain.

***Corresponding authors:**

Email: mariajesus.ramos@uclm.es; Phone: +34926295300 Ext. 6348

Email: julian.rodriguez@uclm.es; Phone: +34926295300 Ext. 3462

**Table of contents**


1. **Characterization of synthesized products.** ........ 3
2. ***In vitro* viability assay** ........ 17


**Supplementary Figures**


**Figure S1.** $^{1}$H NMR of **ABrP**. ........ 3
**Figure S2.** $^{13}$C NMR of **ABrP**. ........ 3
**Figure S3.** $^{1}$H NMR of **5-FUDA**. ........ 4
**Figure S4.** $^{13}$C NMR of **5-FUDA**. ........ 4
**Figure S5.** DEPT of **5-FUDA**. ........ 5
**Figure S6.** $^{19}$F NMR of **5-FUDA**. ........ 5
**Figure S7.** FT-IR of **5-FUDA**. ........ 6
**Figure S8.** MALDI-TOF MS of **5-FUDA**. ........ 6
**Figure S9.** $^{1}$H NMR of **7-POC**. ........ 7
**Figure S10.** $^{13}$C NMR of **7-POC**. ........ 7
**Figure S11.** APT of **7-POC**. ........ 8
**Figure S12.** FT-IR of **7-POC**. ........ 8
**Figure S13.** $^{1}$H NMR of **CP1**. ........ 9
**Figure S14.** $^{13}$C NMR of **CP1**. ........ 9
**Figure S15.** DEPT of **CP1**. ........ 10
**Figure S16.** $^{19}$F NMR of **CP1**. ........ 10
**Figure S17.** FT-IR of **CP1**. ........ 11
**Figure S18.** MALDI-TOF MS of **CP1**. ........ 11
**Figure S19.** $^{1}$H NMR of **4-POC**. ........ 12
**Figure S20.** $^{13}$C NMR of **4-POC**. ........ 12
**Figure S21.** APT of **4-POC**. ........ 13
**Figure S22.** FT-IR of **4-POC**. ........ 13
**Figure S23.** $^{1}$H NMR of **CP2**. ........ 14
**Figure S24.** $^{13}$C NMR of **CP2**. ........ 14
**Figure S25.** DEPT of **CP2** ........ 15
**Figure S26.** $^{19}$F NMR of **CP2**. ........ 15
**Figure S27.** FT-IR of **CP2**. ........ 16
**Figure S28.** MALDI-TOF MS of **CP2**. ........ 16
**Figure S29.** Microscopic images (magnification 10×) of PANC-1 cells in MTT assays. ........ 17

# 1. Characterization of synthesized products.

## 1.1. 1-Azido-5-bromopentane (ABrP).

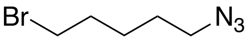


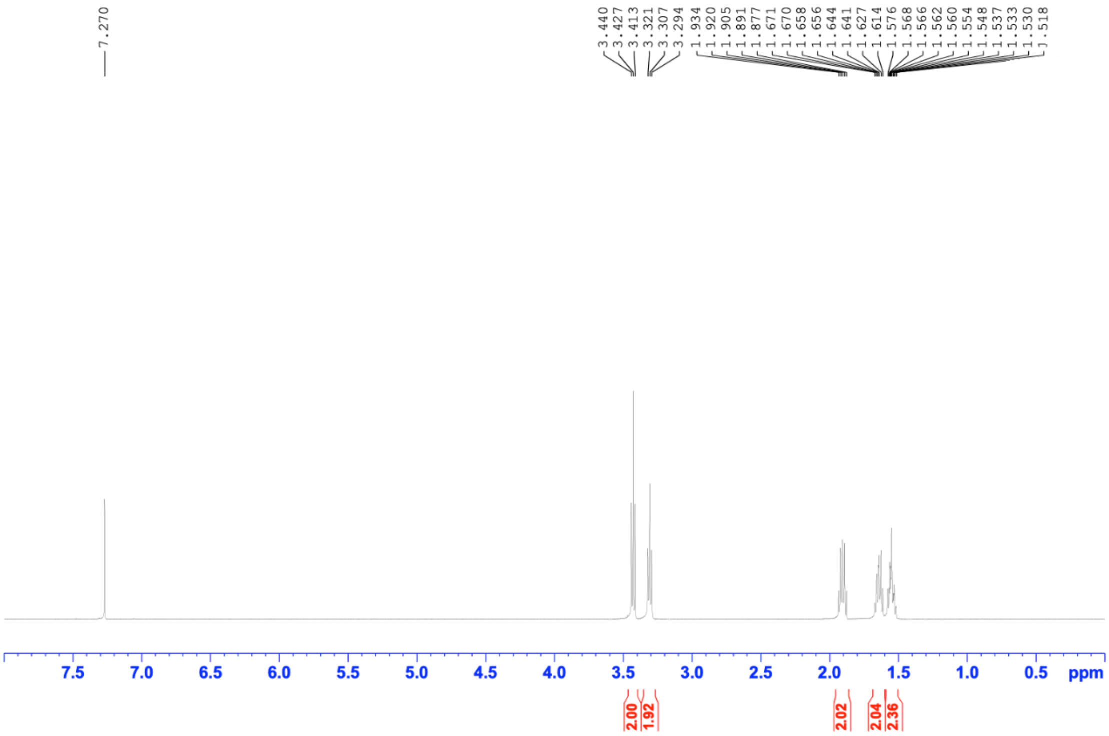


***Figure S1.*** *$^1$H NMR (CDCl$_3$, 500 MHz) of **ABrP**.*

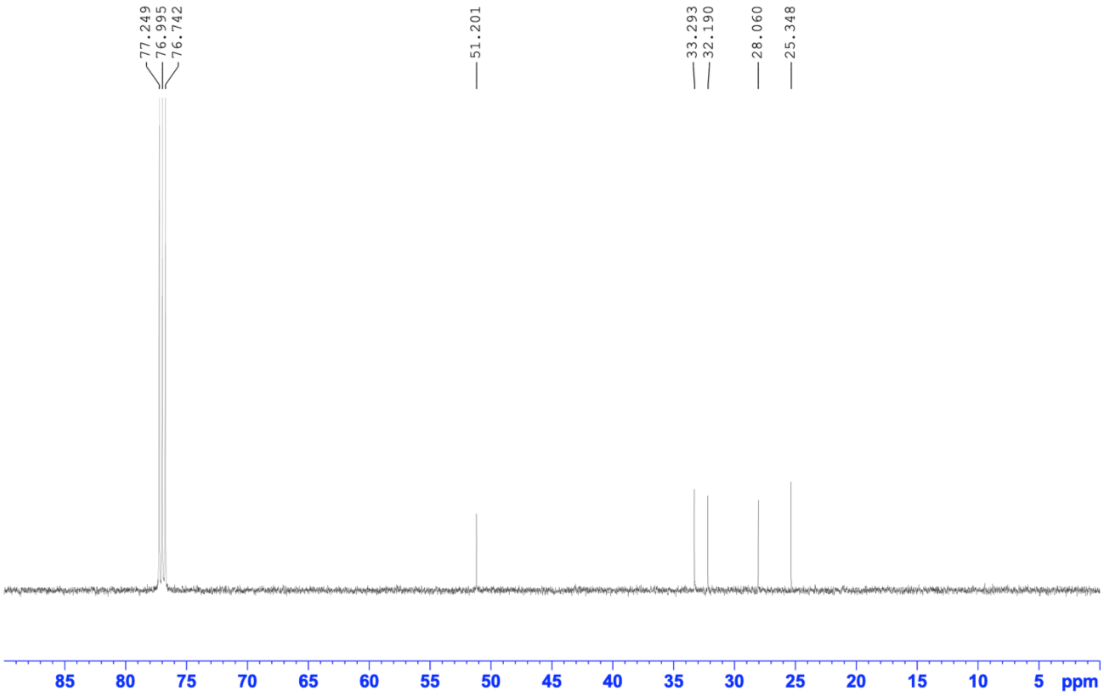


***Figure S2.*** *$^{13}$C NMR (CDCl$_3$, 125 MHz) of **ABrP**.*

## 1.2. 1,3-Bis(5-azidopentyl)-5-fluorouracil (5-FUDA).

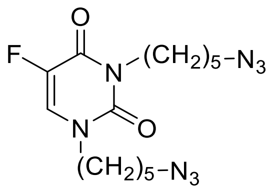


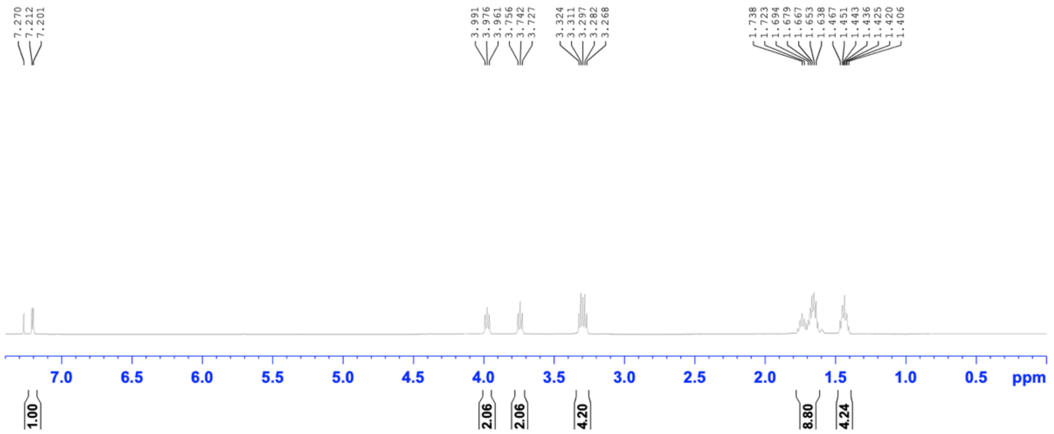


***Figure S3.*** *$^{1}$H NMR (CDCl$_3$, 500 MHz) of* ***5-FUDA****.*

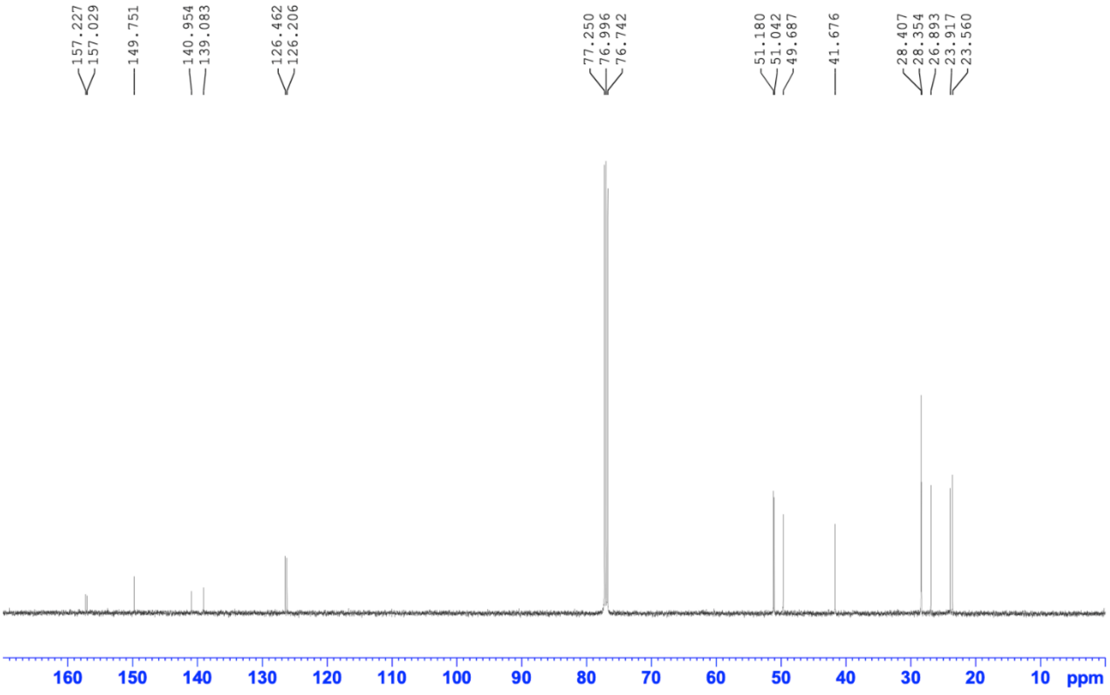


***Figure S4.*** *$^{13}$C NMR (CDCl$_3$, 125 MHz) of* ***5-FUDA****.*

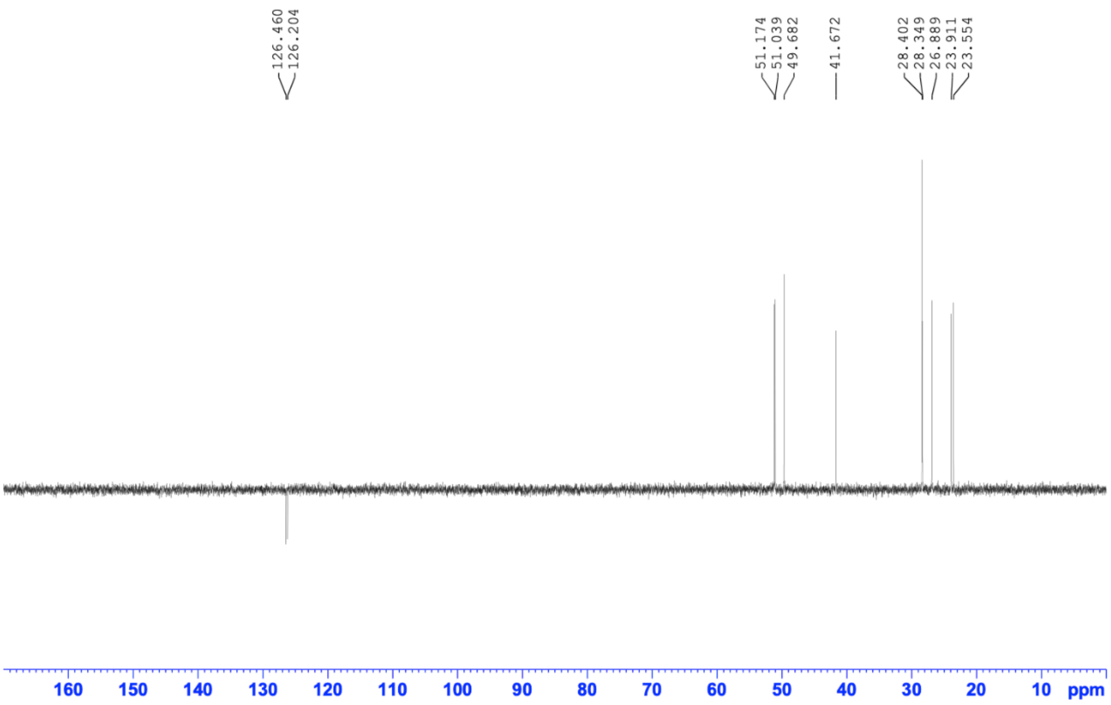


***Figure S5.*** *DEPT ($CDCl_3$, 125 MHz) of* ***5-FUDA****.*

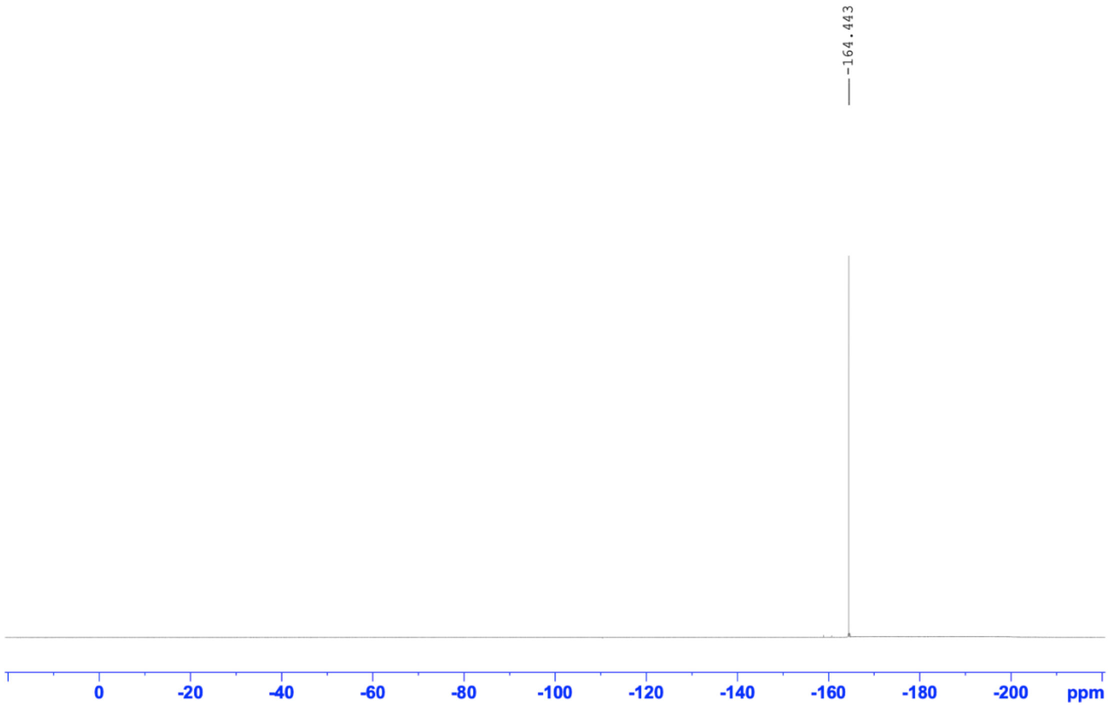


***Figure S6.*** *$^{19}F$ NMR ($CDCl_3$, 471 MHz) of* ***5-FUDA****.*

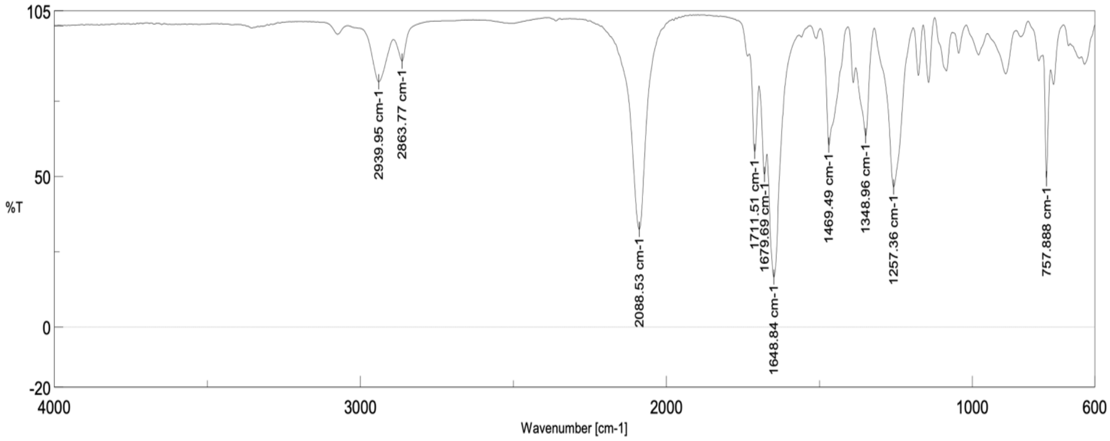


***Figure S7.*** *FT-IR (ATR) of* ***5-FUDA***.

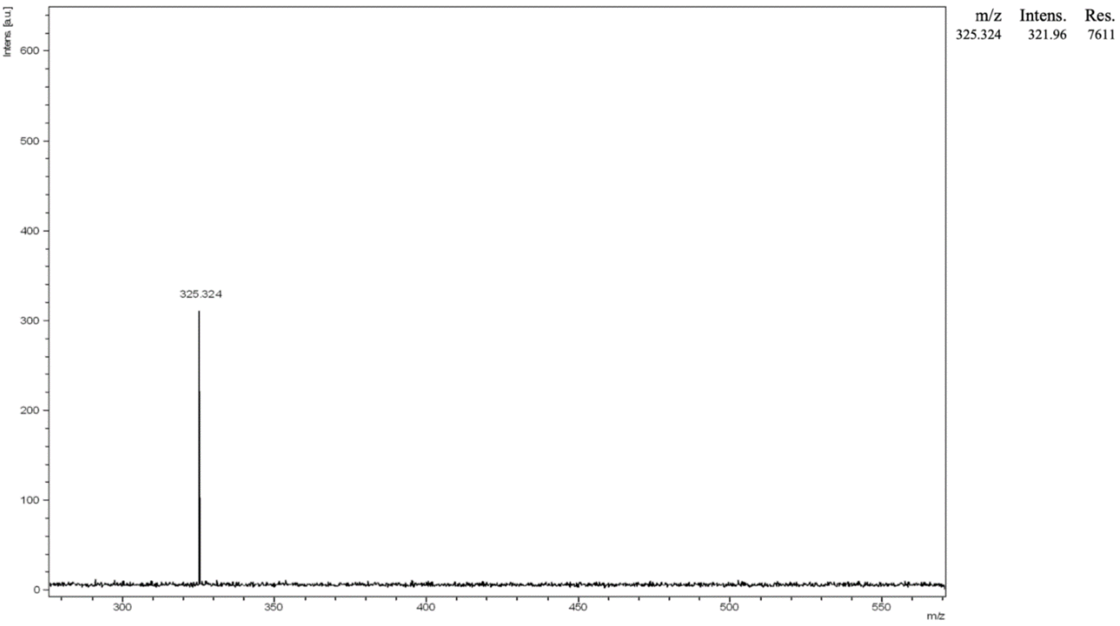


***Figure S8.*** *MALDI-TOF MS (dithranol) of* ***5-FUDA***.

## 1.3. 7-Propargyloxycoumarine (7-POC).

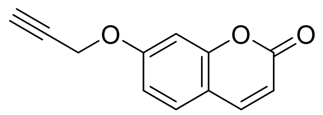


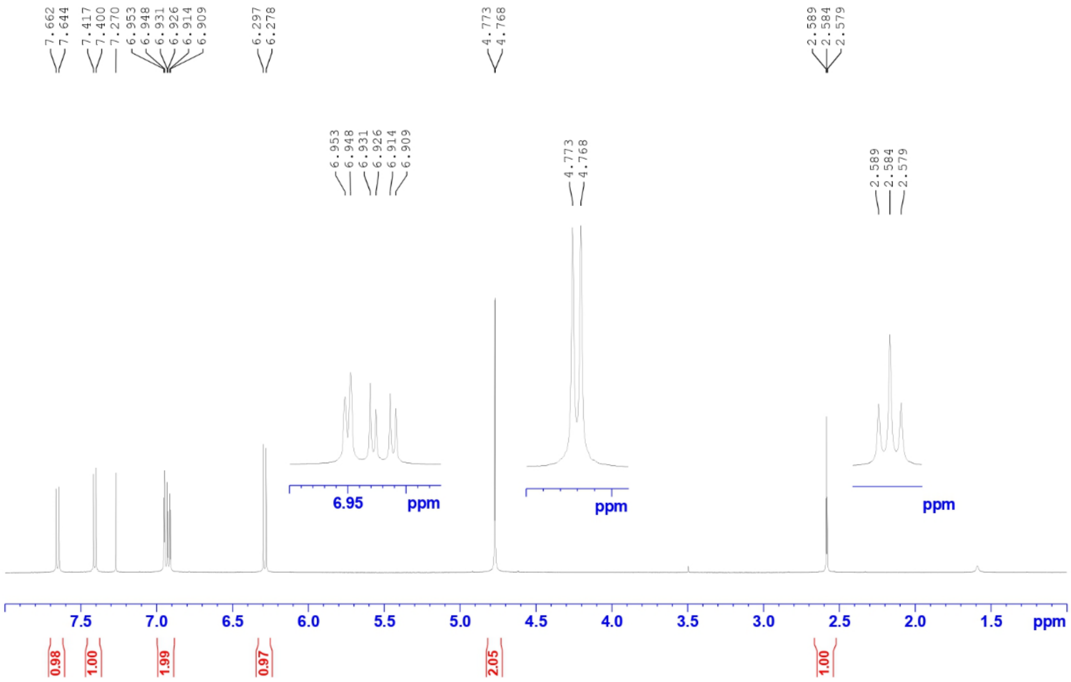


***Figure S9.*** *$^{1}$H NMR (CDCl₃, 500 MHz) of* ***7-POC****.*

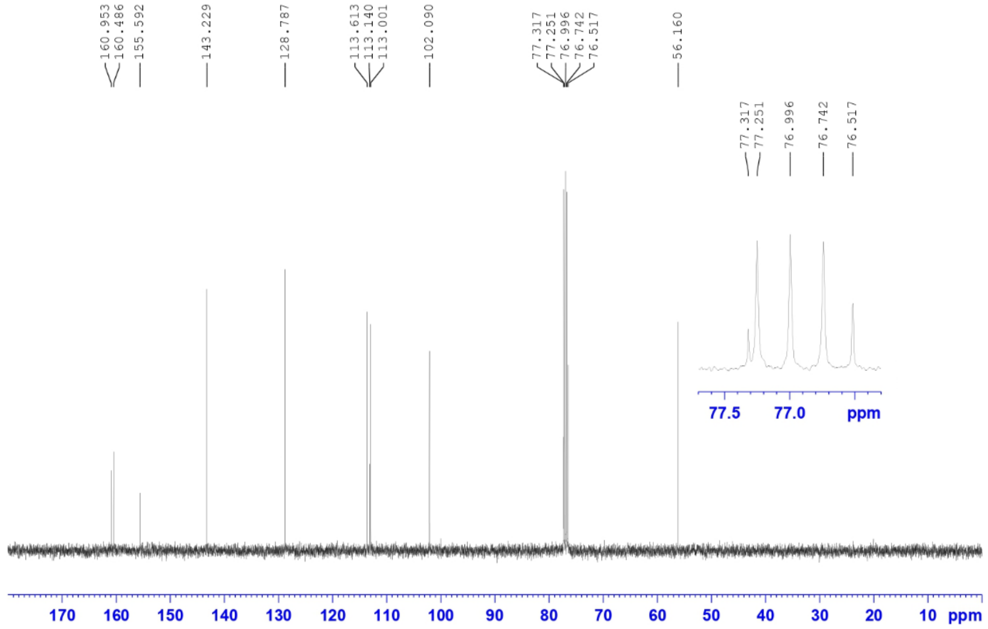


***Figure S10.*** *$^{13}$C NMR (CDCl₃, 125 MHz) of* ***7-POC****.*

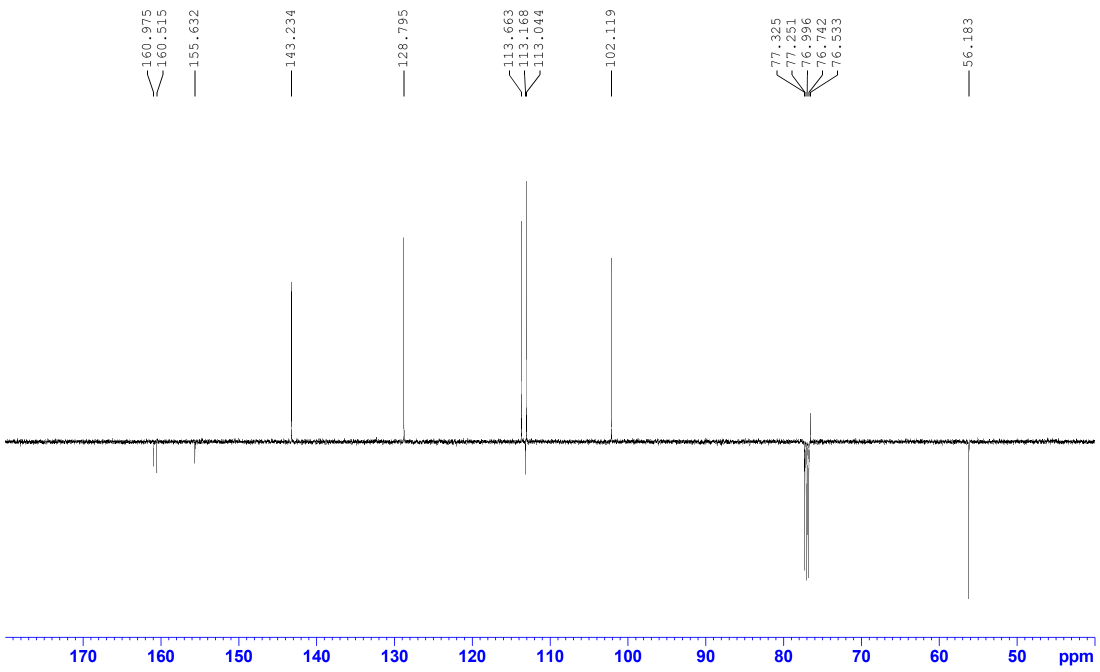


***Figure S11.*** *APT (CDCl₃, 125 MHz) of 7-**POC**.*

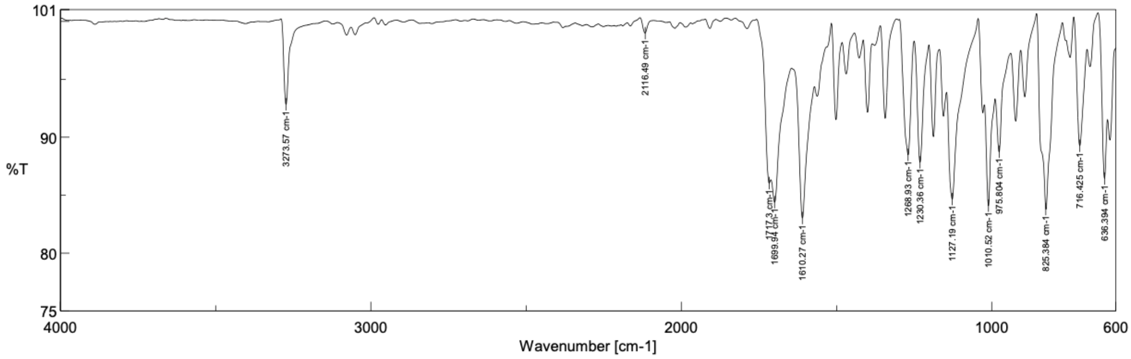


***Figure S12.*** *FT-IR (ATR) of 7-**POC**.*

## 1.4. Click product CP1.

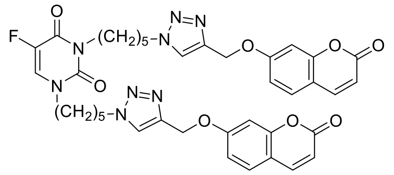

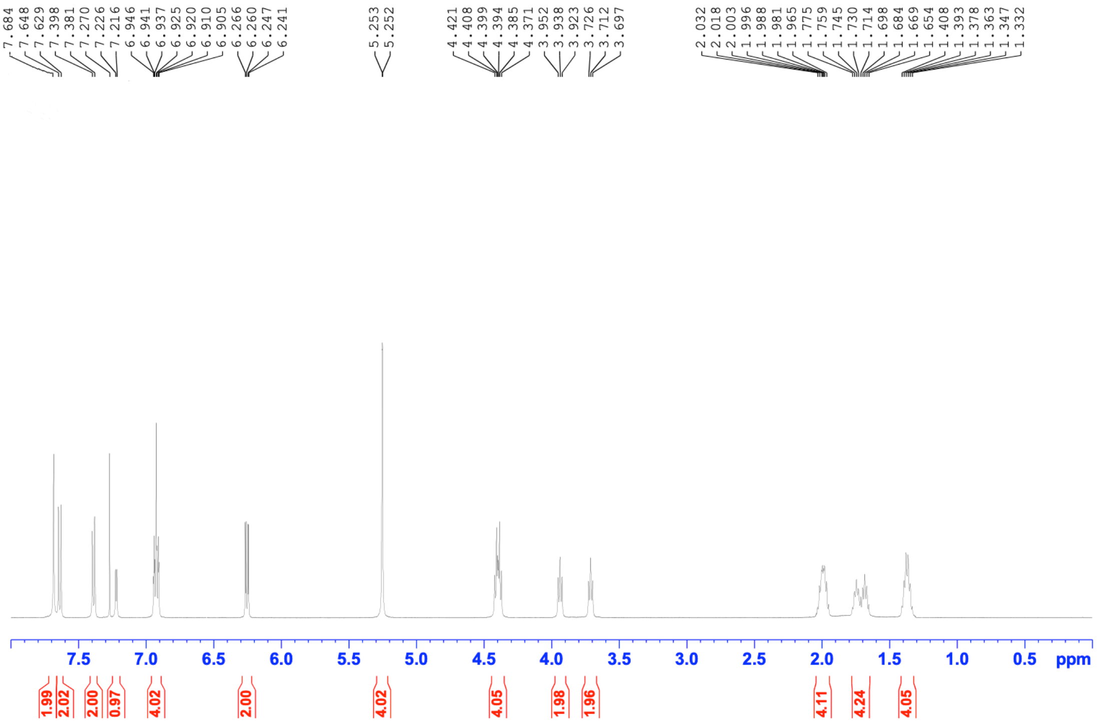


***Figure S13.*** *$^{1}$H NMR ($CDCl_3$, 500 MHz) of* ***CP1***.

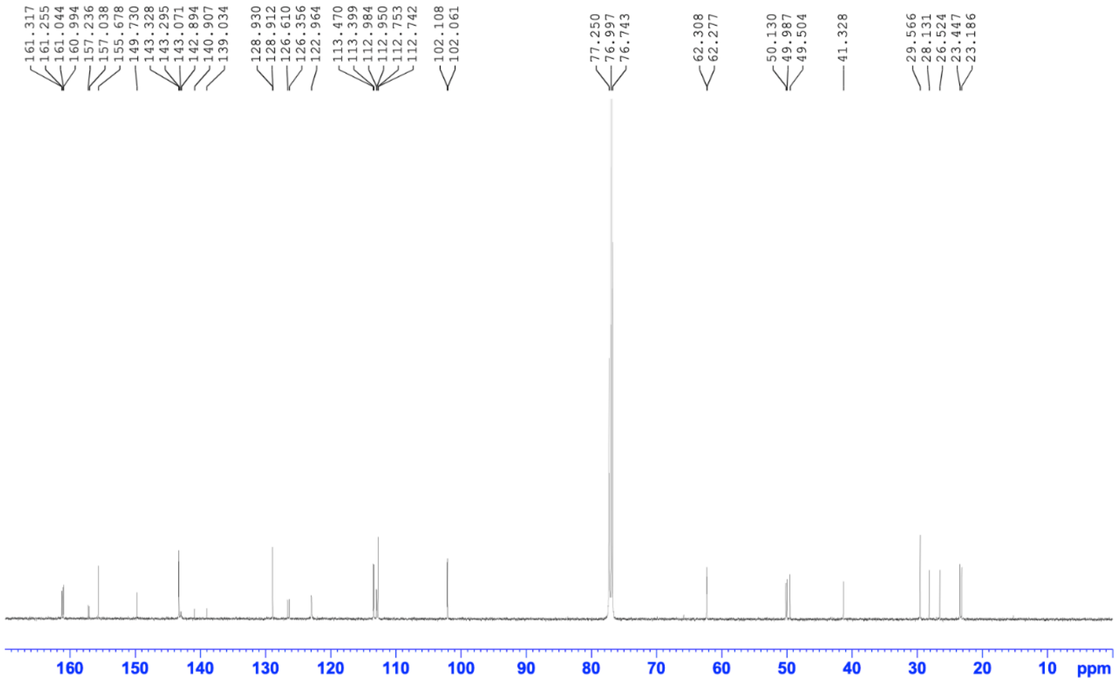


***Figure S14.*** *$^{13}$C NMR ($CDCl_3$, 125 MHz) of* ***CP1***.

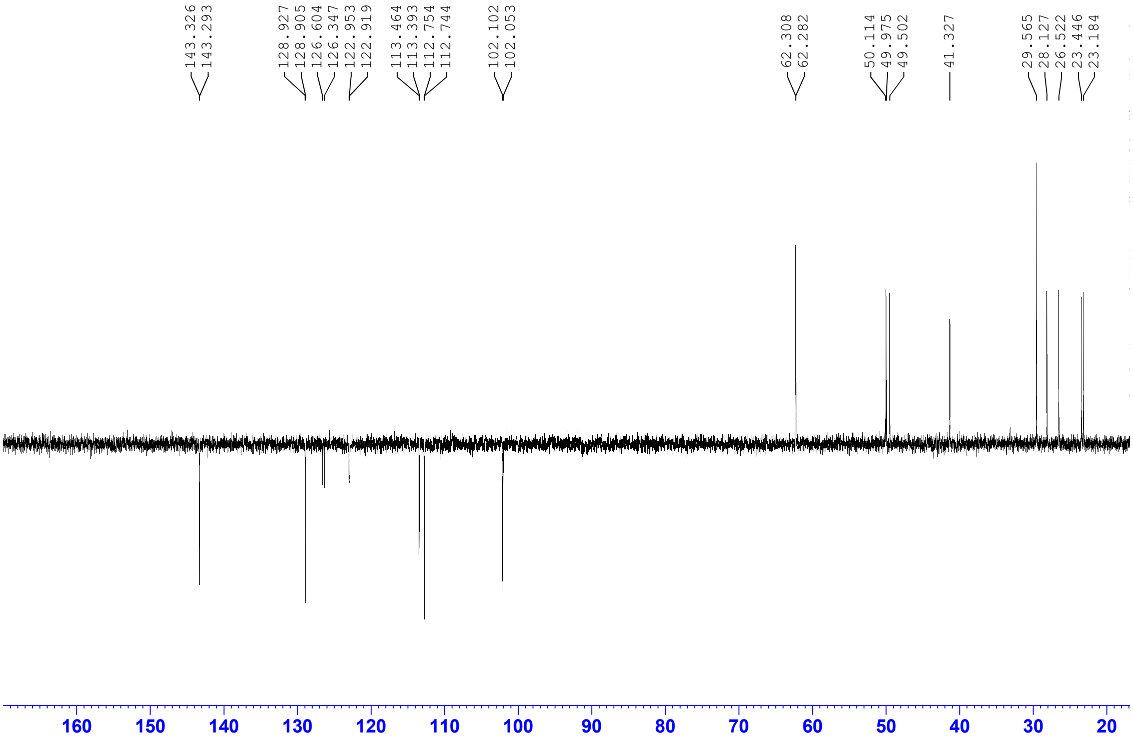


***Figure S15.*** *DEPT ($CDCl_3$, 125 MHz) of* ***CP1****.*

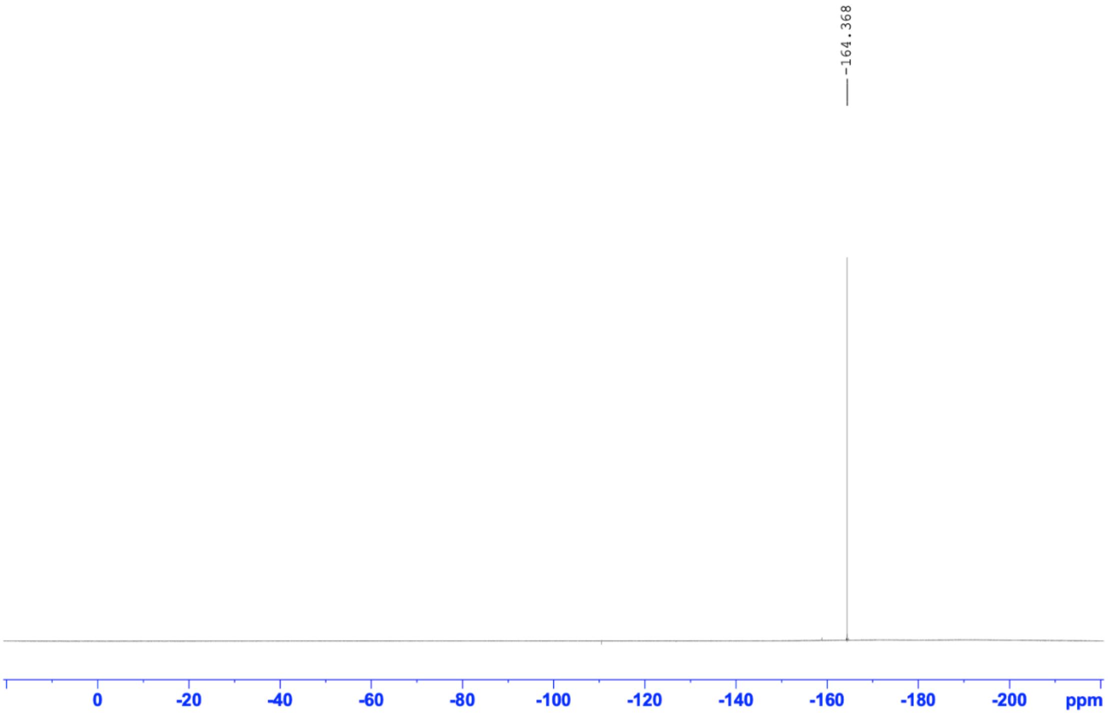


***Figure S16.*** *$^{19}$F NMR ($CDCl_3$, 471 MHz) of* ***CP1****.*

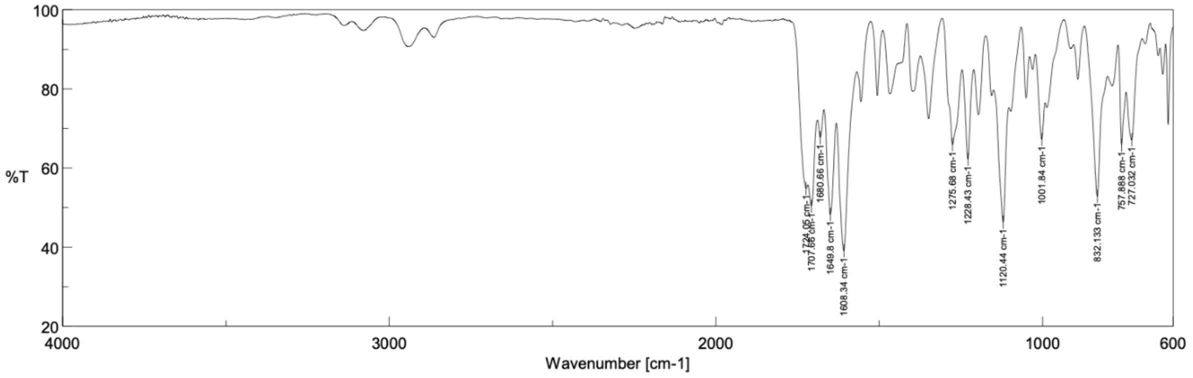


***Figure S17.*** *FT-IR (ATR) of* ***CP1****.*

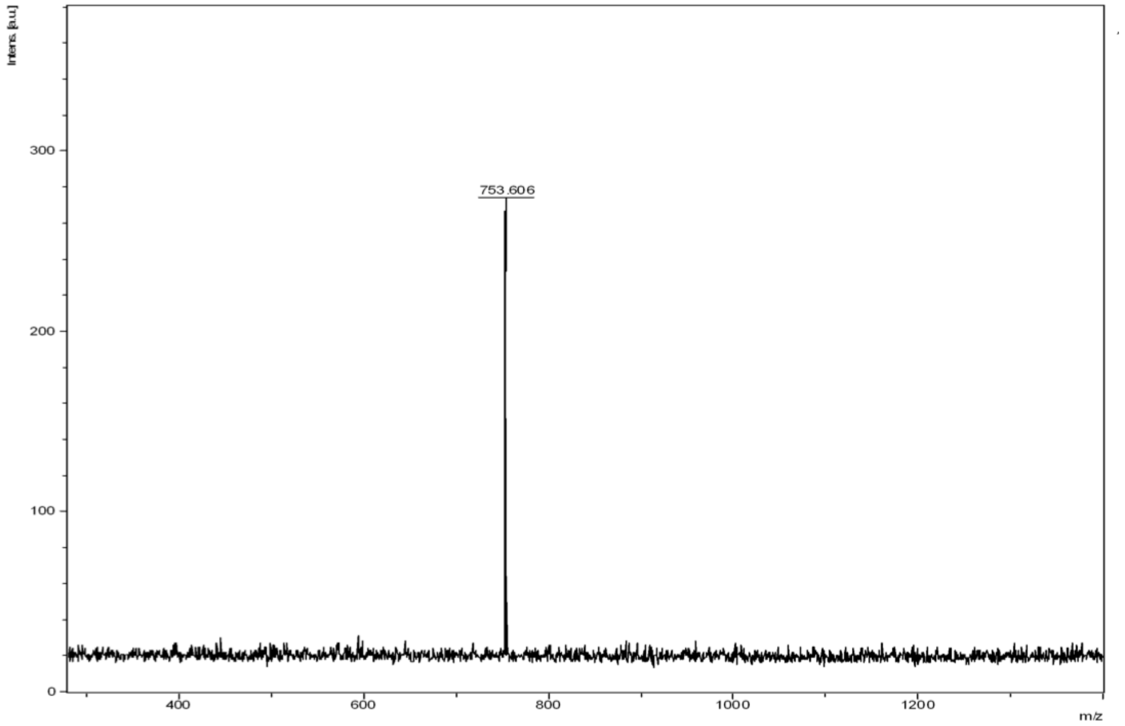


***Figure S18.*** *MALDI-TOF MS (dithranol) of* ***CP1****.*

## 1.5. 4-Propargyloxycoumarin (4-POC).

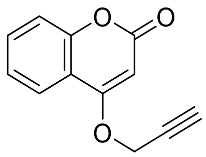

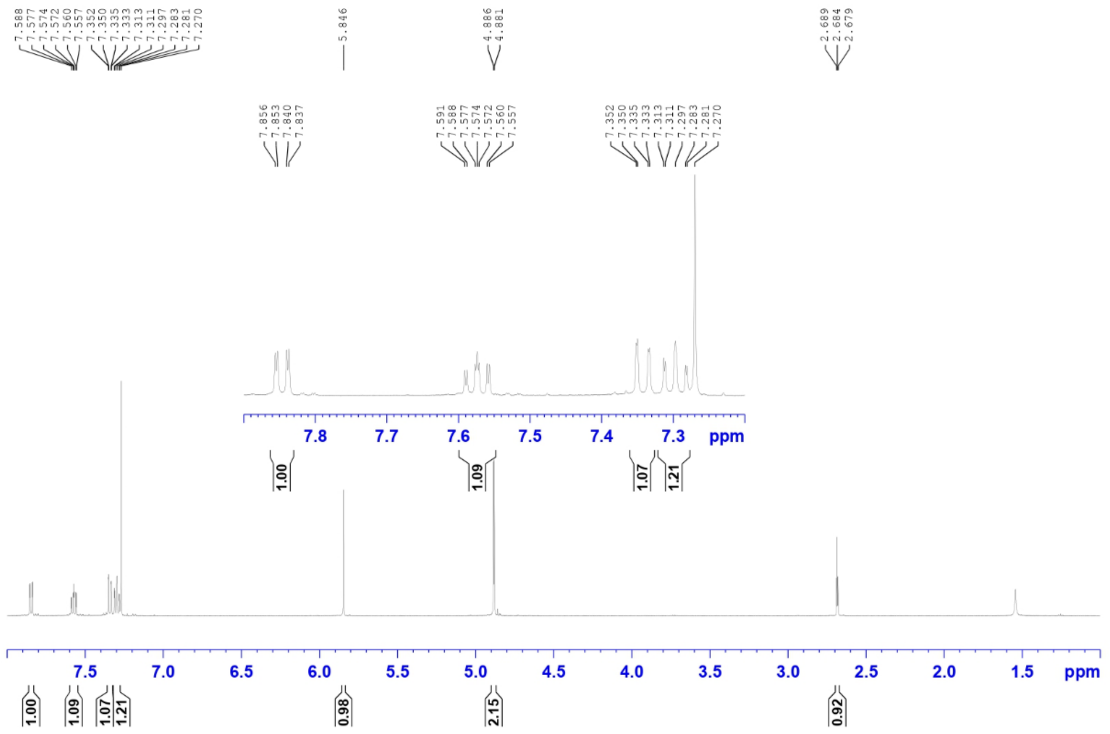

***Figure S19.*** *$^{1}H$ NMR ($CDCl_3$, 500 MHz) of* ***4-POC***.

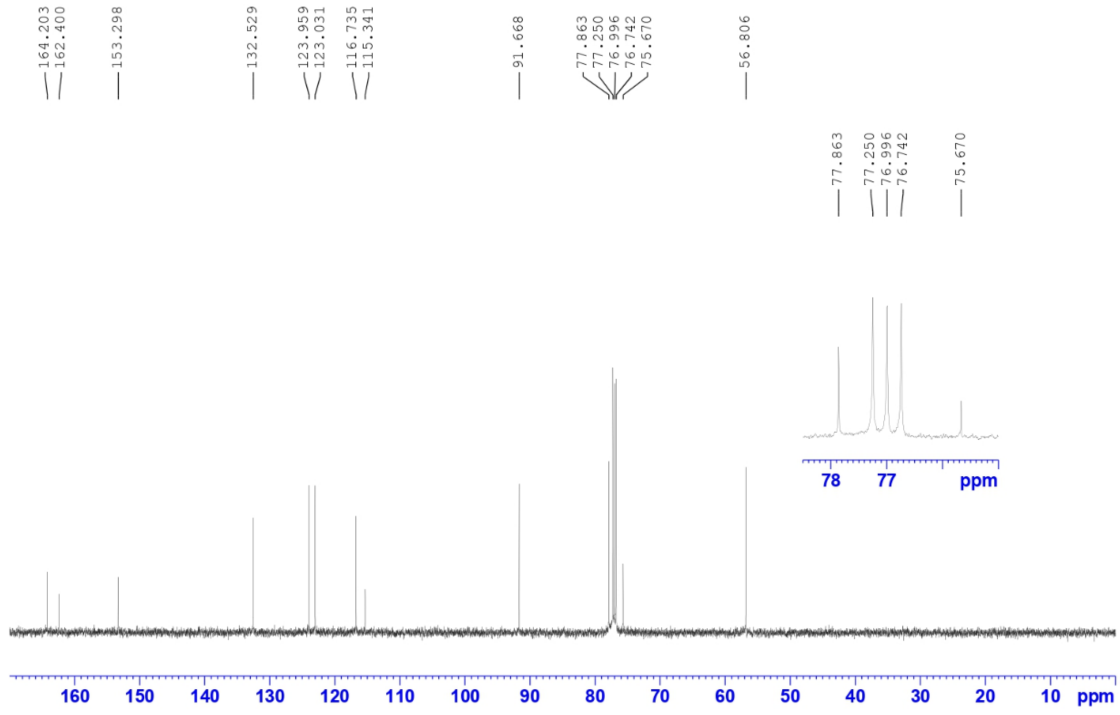

***Figure S20.*** *$^{13}C$ NMR ($CDCl_3$, 125 MHz) of* ***4-POC***.

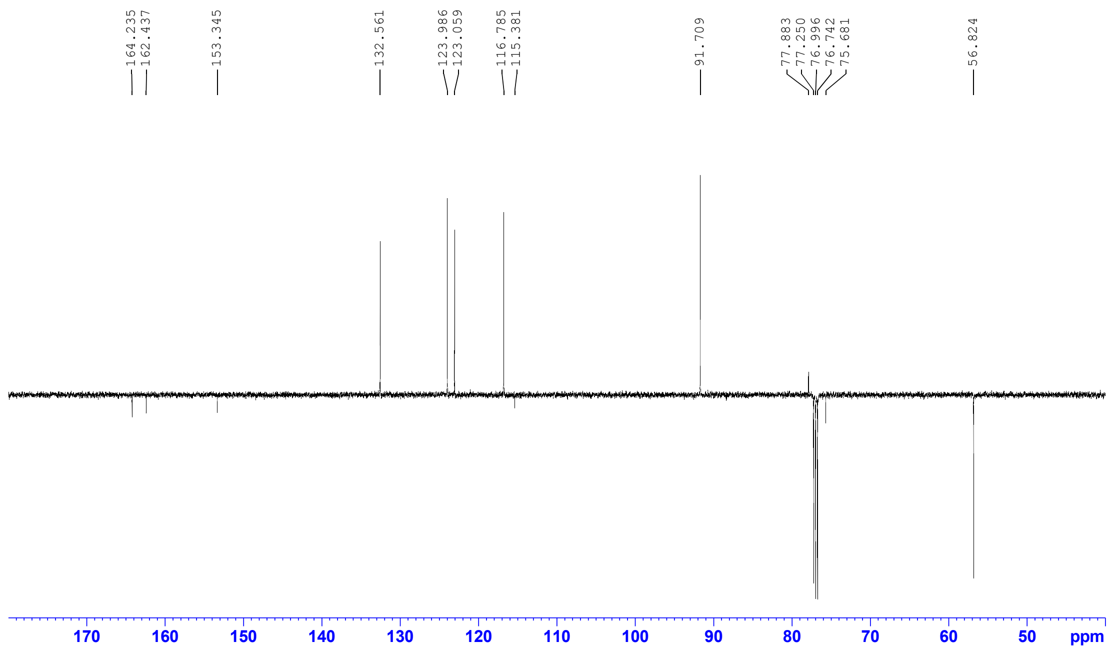


***Figure S21.*** *APT (CDCl$_3$, 125 MHz) of* ***4-POC****.*

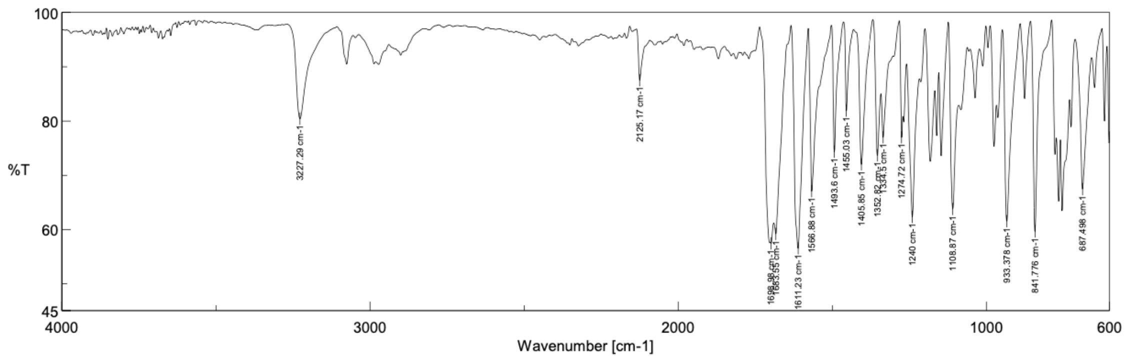


***Figure S22.*** *FT-IR (ATR) of* ***4-POC****.*

## 1.6. Click product CP2.

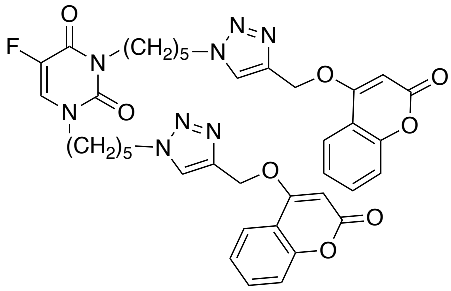

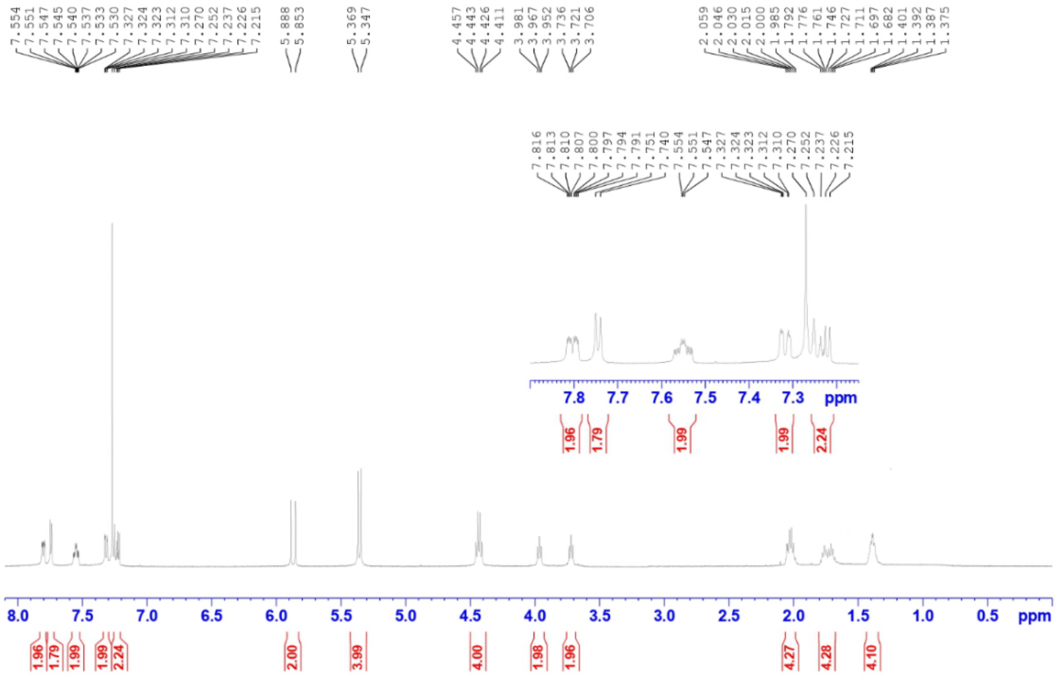

***Figure S23.*** *$^{1}$H NMR ($CDCl_3$, 500 MHz) of* ***CP2****.*

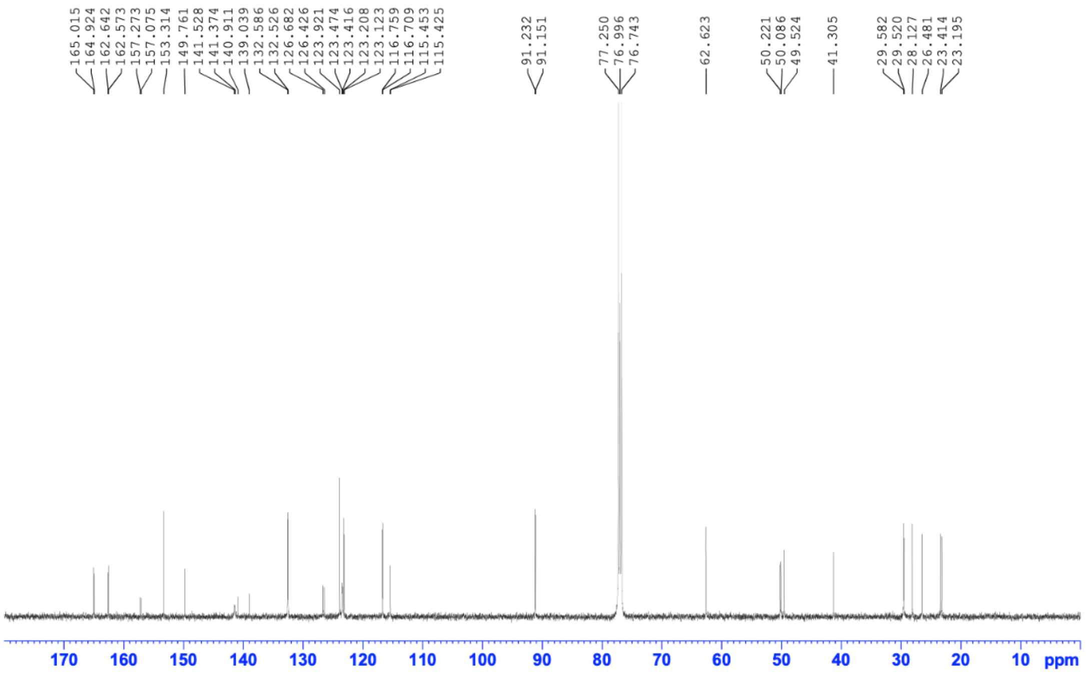

***Figure S24.*** *$^{13}$C NMR ($CDCl_3$, 125 MHz) of* ***CP2****.*

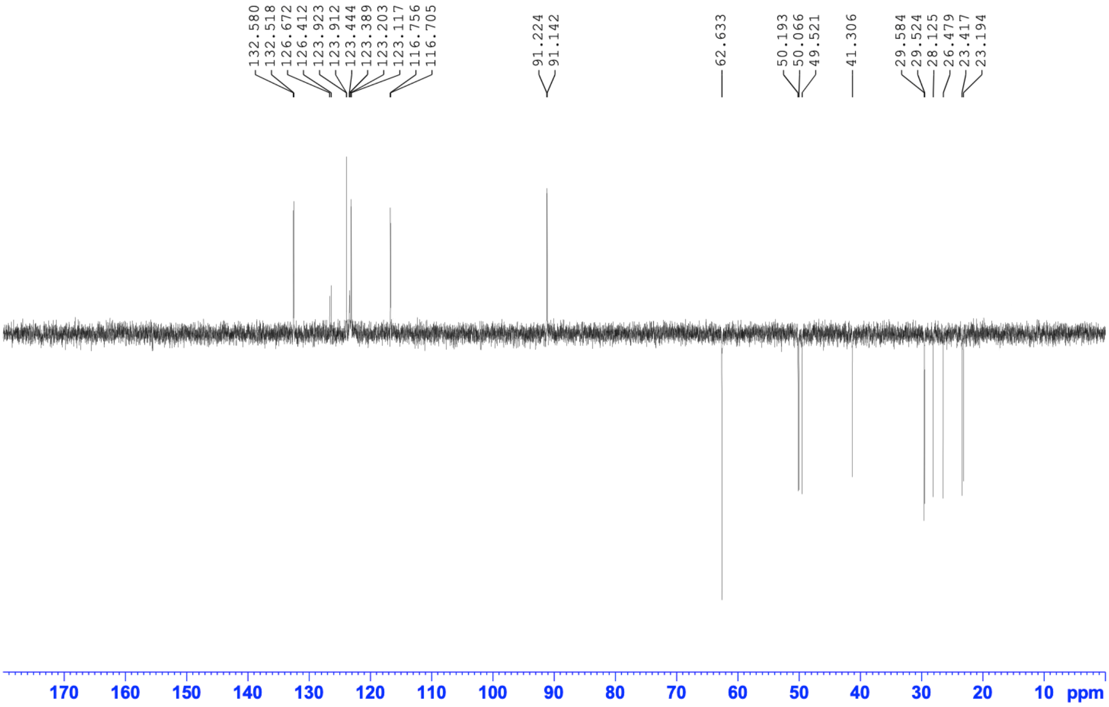


***Figure S25.*** *DEPT ($CDCl_3$, 125 MHz) of* ***CP2****.*

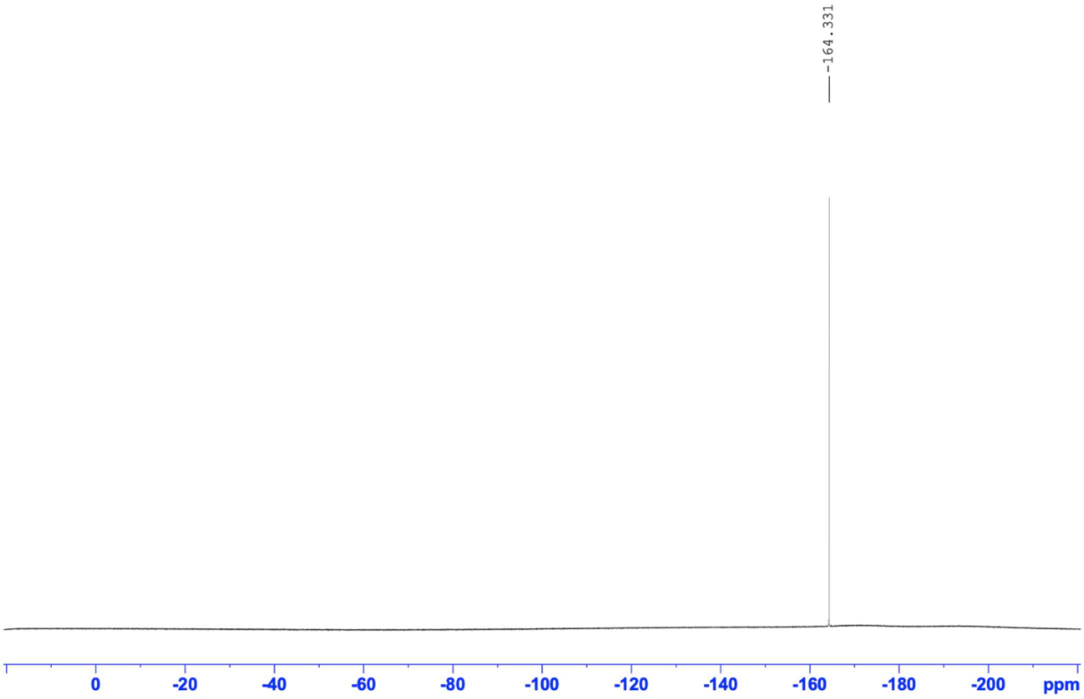


***Figure S26.*** *$^{19}F$ NMR ($CDCl_3$, 471 MHz) of* ***CP2****.*

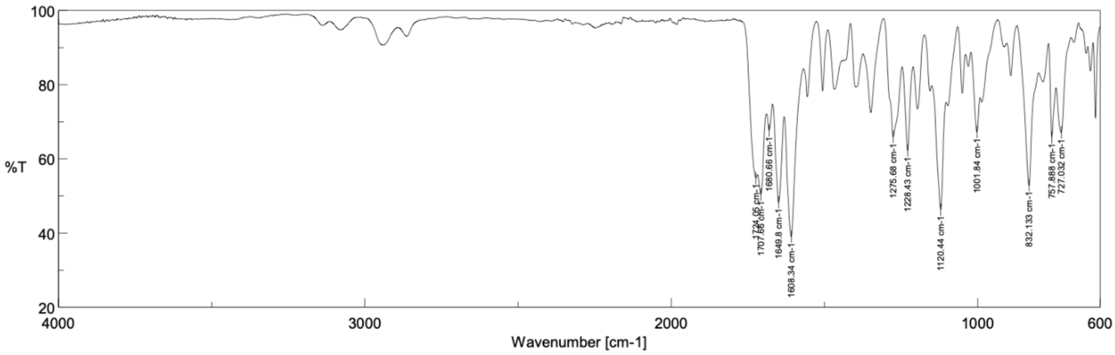


***Figure S27.*** *FT-IR (ATR) of* ***CP2****.*

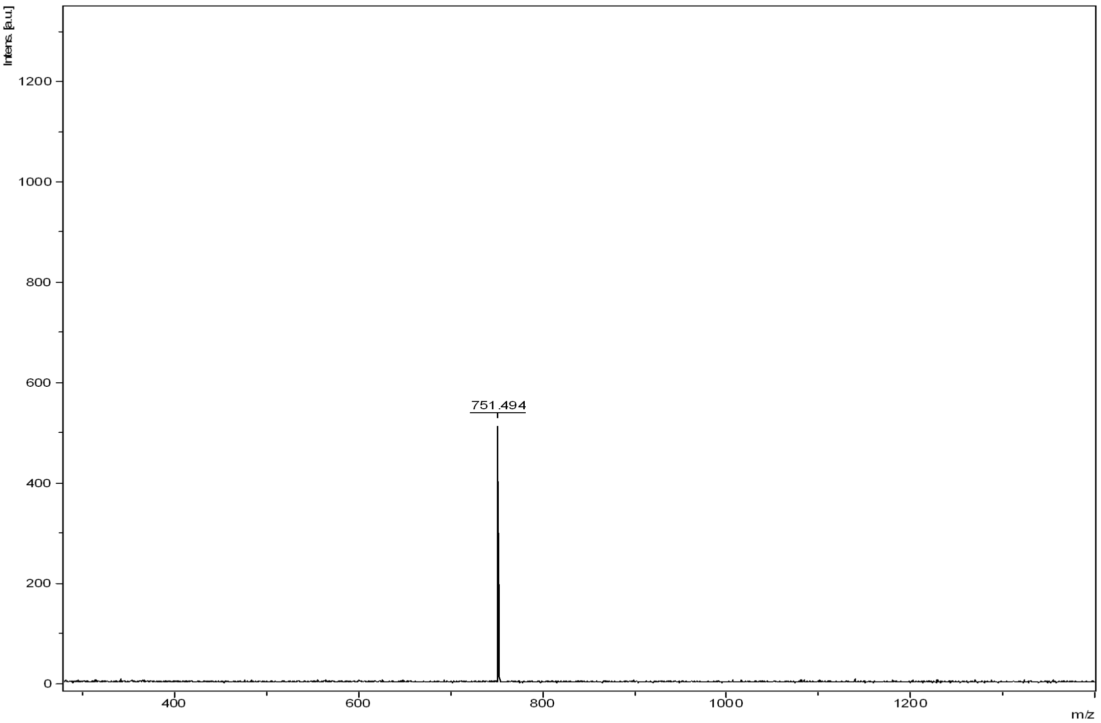


***Figure S28.*** *MALDI-TOF MS (dithranol) of* ***CP2****.*

## 2. *In vitro* viability assay.

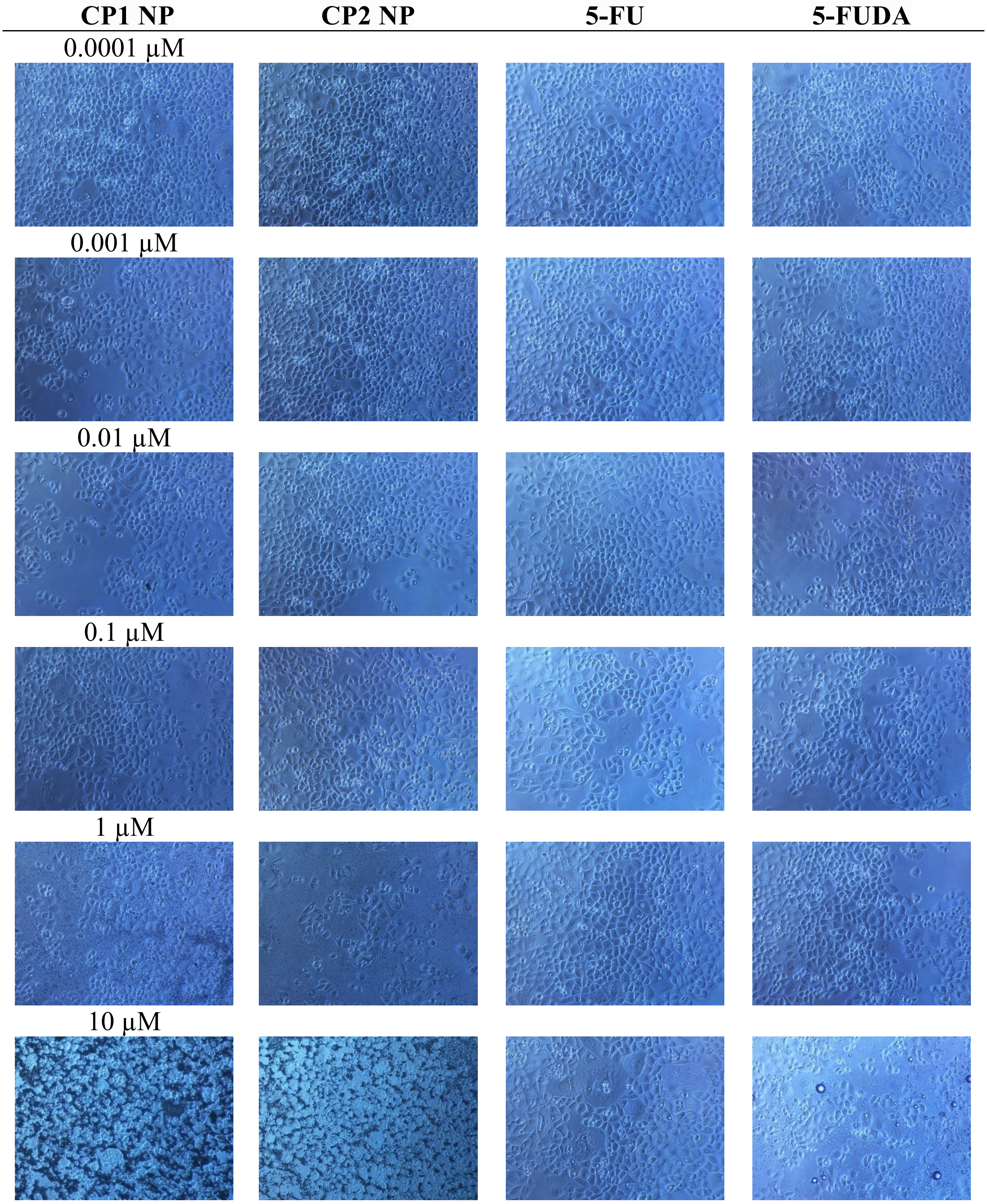


***Figure S29.*** *Microscopic images (magnification 10×) of PANC-1 cells after treatment with* ***5-FU****,* ***5-FUDA****,* ***CP1****, and* ***CP2*** *NPs in MTT assays at different concentrations.*
